# Worldwide Genetic Structure Elucidates the Eurasian Origin and Invasion Pathways of *Dothistroma septosporum*, Causal Agent of Dothistroma Needle Blight

**DOI:** 10.3390/jof7020111

**Published:** 2021-02-03

**Authors:** Martin S. Mullett, Rein Drenkhan, Kalev Adamson, Piotr Boroń, Anna Lenart-Boroń, Irene Barnes, Michal Tomšovský, Zuzana Jánošíková, Katarína Adamčíková, Emília Ondrušková, Valentin Queloz, Barbara Piškur, Dmitry L. Musolin, Kateryna Davydenko, Margarita Georgieva, Sophie Schmitz, Audrius Kačergius, Luisa Ghelardini, Jelena Kranjec Orlović, Michael Müller, Funda Oskay, Tine Hauptman, Ágnes Halász, Svetlana Markovskaja, Halvor Solheim, Martti Vuorinen, Renate Heinzelmann, Richard C. Hamelin, Adam Konečný

**Affiliations:** 1 Phytophthora Research Centre, Faculty of Forestry and Wood Technology, Department of Forest Protection and Wildlife Management, Mendel University in Brno, Zemědělská 3, 61300 Brno, Czech Republic; michal.tomsovsky@mendelu.cz; 2 Institute of Forestry and Rural Engineering, Estonian University of Life Sciences, 51006 Tartu, Estonia; rein.drenkhan@emu.ee (R.D.); kalev.adamson@emu.ee (K.A.); 3 Department Forest Ecosystems Protection, University of Agriculture in Kraków, 31-425 Kraków, Poland; piotr.boron@urk.edu.pl; 4 Department of Microbiology and Biomonitoring, University of Agriculture in Kraków, 30-059 Kraków, Poland; anna.lenart-boron@urk.edu.pl; 5 Department of Biochemistry, Genetics and Microbiology, Forestry and Agricultural Biotechnology Institute (FABI), University of Pretoria (UP), Pretoria 0002, South Africa; irene.barnes@fabi.up.ac.za; 6 Department of Plant Pathology and Mycology, Institute of Forest Ecology SAS, 949 01 Nitra, Slovakia; janosikova@ife.sk (Z.J.); katarina.adamcikova@ife.sk (K.A.); ondruskova@ife.sk (E.O.); 7 Swiss Federal Research Institute WSL, Swiss Forest Protection, 8903 Birmensdorf, Switzerland; valentin.queloz@wsl.ch; 8 Department of Forest Protection, Slovenian Forestry Institute, Večna pot 2, SI-1000 Ljubljana, Slovenia; barbara.piskur@gozdis.si (B.P.); tine.hauptman@bf.uni-lj.si (T.H.); 9 Department of Forest Protection, Wood Science and Game Management, Saint Petersburg State Forest Technical University, 194021 Saint Petersburg, Russia; musolin@gmail.com; 10 Department of Forest Protection, Ukrainian Research Institute of Forestry & Forest Melioration, 61024 Kharkiv, Ukraine; kateryna.davydenko74@gmail.com; 11 Department of Forest Mycology and Plant Pathology, Swedish University of Agricultural Sciences, 75007 Uppsala, Sweden; 12 Department of Forest Entomology, Phytopathology and Game Fauna, Forest Research Institut—Bulgarian Academy of Sciences, 1756 Sofia, Bulgaria; margaritageorgiev@gmail.com; 13 Walloon Agricultural Research Centre, Department of Life Sciences, B-5030 Gembloux, Belgium; s.schmitz@cra.wallonie.be; 14 Vokė Branch of Lithuanian Research Centre for Agriculture and Forestry, LT-02232 Vilnius, Lithuania; audrius.kacergius@lammc.lt; 15 Department of Agricultural, Food, Environmental and Forest Sciences and Technologies (DAGRI), University of Florence, 50144 Firenze, Italy; luisa.ghelardini@unifi.it; 16 Institute for Sustainable Plant Protection (IPSP), National Research Council of Italy (CNR), 50019 Sesto Fiorentino, Italy; 17 Faculty of Forestry, University of Zagreb, 10002 Zagreb, Croatia; jkranjec@sumfak.hr; 18 Natural Resources Institute Finland (Luke), Bioeconomy and Environment, P.O. Box 2, FI-00791 Helsinki, Finland; micms.muller@gmail.com; 19 Faculty of Forestry, Çankırı Karatekin University, 18200 Çankırı, Turkey; fundaoskay@karatekin.edu.tr; 20 Department of Forestry and Renewable Forest Resources, Biotechnical Faculty, University of Ljubljana, Večna pot 83, SI-1000 Ljubljana, Slovenia; 21 Plant Health Diagnostic National Reference Laboratory, National Food Chain Safety Office, H-1118 Budapest, Hungary; halasza@nebih.gov.hu; 22 Nature Research Centre, Institute of Botany, Žaliųjų Ežerų Str. 49, LT-08406 Vilnius, Lithuania; svetlana.markovskaja@gamtc.lt; 23 Norwegian institute of Bioeconomy, P.O. Box 115, N-1431 Ås, Norway; halvor.solheim@nibio.no; 24 Natural Resources Institute (LUKE), FI-77600 Suonenjoki, Finland; martti.vuorinen@luke.fi; 25 Department of Forest and Conservation Sciences, Faculty of Forestry, The University of British Columbia, 2424 Main Mall, Vancouver, BC V6T 1Z4, Canada; renate.heinzelmann@ubc.ca (R.H.); richard.hamelin@ubc.ca (R.C.H.); 26 Institut de Biologie Intégrative et des Systèmes (IBIS), Université Laval/Pavillon Charles-Eugène Marchand, 1030 Avenue de la Médecine, Québec City, QC G1V 0A6, Canada; 27 Department of Botany and Zoology, Faculty of Science, Masaryk University, 61137 Brno, Czech Republic; akonecny@sci.muni.cz

**Keywords:** *Mycosphaerella pini*, biogeography, ABC, DNB, global spread, introduction pathways, invasive pathogen, global spread

## Abstract

*Dothistroma septosporum*, the primary causal agent of Dothistroma needle blight, is one of the most significant foliar pathogens of pine worldwide. Its wide host and environmental ranges have led to its global success as a pathogen and severe economic damage to pine forests in many regions. This comprehensive global population study elucidated the historical migration pathways of the pathogen to reveal the Eurasian origin of the fungus. When over 3800 isolates were examined, three major population clusters were revealed: North America, Western Europe, and Eastern Europe, with distinct subclusters in the highly diverse Eastern European cluster. Modeling of historical scenarios using approximate Bayesian computation revealed the North American cluster was derived from an ancestral population in Eurasia. The Northeastern European subcluster was shown to be ancestral to all other European clusters and subclusters. The Turkish subcluster diverged first, followed by the Central European subcluster, then the Western European cluster, which has subsequently spread to much of the Southern Hemisphere. All clusters and subclusters contained both mating-types of the fungus, indicating the potential for sexual reproduction, although asexual reproduction remained the primary mode of reproduction. The study strongly suggests the native range of *D. septosporum* to be in Eastern Europe (i.e., the Baltic and Western Russia) and Western Asia.

## 1. Introduction

Dothistroma needle blight (DNB) is one of the most important and damaging diseases of pines worldwide, affecting over 109 Pinaceae taxa [1]. The disease causes foliar necrosis, premature needle cast, reduction in growth, and in extreme cases, tree death [2]. DNB causes large economic losses (e.g., NZD $24 million per year in New Zealand [3] and GBP £8.6 million per year in the UK [4]) due to timber losses from forest plantations, but also negatively affects the landscape and recreational value of forests and the esthetic value of ornamentals, with non-timber losses estimated at GBP £50 million per year in the UK alone [5].

The disease is caused by two species: *Dothistroma septosporum* (Doroguine) Morelet and *D. pini* Hulbary, which are only distinguishable using molecular methods [6]. *Dothistroma septosporum* has a worldwide distribution, being present on all continents where available hosts grow in habitats ranging from tropical to sub-arctic on a large host range [1]. On the other hand, *D. pini* has a much more restricted host and geographical range, being present in parts of east–central USA and a limited number of European locations [1,7].

DNB achieved notoriety in the 1950s and 1960s in the Southern Hemisphere, where it caused and continues to cause extensive damage to non-native pine plantations [1,8]. Since the 1990s, however, DNB has increased in incidence and severity in the Northern Hemisphere, with severe largescale outbreaks occurring in Canada, the UK, and France, and with the disease being reported for the first time from much of central and northern Europe [1]. Reasons for this increase in incidence and severity in the last 30 years has been ascribed to various aspects of the disease triangle: host genotype, pathogen genotype and environment. A number of studies have concluded that contributing factors to this disease escalation are increased host availability through the expansion of plantation forests, often of more susceptible non-native hosts, and changing climatic conditions, particularly above-average precipitation [1,9,10,11]. However, a comprehensive global population study to investigate the role of pathogen genotype and population has not been conducted to date.

DNB has been observed in some regions for over 100 years, for example, Northeastern France [12], European Russia [13], Ukraine [1], and parts of the USA [14,15,16], with dendrochronological studies indicating its presence in British Columbia, Canada since 1831 [10,17]. However, the origin of the genus and both species is still unknown, with the native area hypothesized to be Central America [14], the Himalayas [18], and parts of North America and Europe [14,19]. Recent genetic studies have not been able to resolve the question of origin, partly due to a lack of substantial samples from Asia and Central America. These studies have, however, shown support for the pathogen being native to parts of both North America and Europe [20,21,22,23].

Individual population studies have shown unexpected levels of diversity in many populations [20,21,22,23,24,25,26,27,28,29]. This diversity indicates that sexual reproduction is more common than previously thought, particularly as the sexual stage (previously known as *Mycosphaerella pini*) has only rarely been observed, and the asexual stage is ubiquitous. Yet, it has also provided support for the hypothesis that the fungus may be native to these areas. However, most of these studies have focused on a single country or region within a country, and it is unclear how these various populations relate to each other, including their pathways of introduction and migration. Only a few studies have included samples from more than one country, and only two [20,21] have explored intercontinental population patterns. Given the global distribution of *D. septosporum,* the objective of this study was to combine isolates from the previous population studies with newly obtained isolates to provide a unified overview and large-scale context of its populations and to investigate the role of pathogen genotype and population in contributing to the increase in serious outbreaks.

Microsatellite data from an unprecedented number of samples were collected from across the worldwide range of *D. septosporum*, encompassing all but three countries from which the pathogen has been reported. The data were modeled using approximate Bayesian computation (ABC), a method used to understand population history, including invasion pathways of fungi, e.g., [30,31]. This likelihood-free technique manages an arbitrary number of populations and samples that are employed in complex evolutionary scenarios and is particularly suited to inferences about introduction histories of invasive species [32]. The specific aims of this study were to (i) tie together and contextualize previous individual population studies by including isolates from previous population studies and newly obtained isolates, (ii) elucidate the phylogeographic relationships of individual regions and populations, (iii) investigate the mating-type and prevalence of sexual recombination in these populations, (iv) determine whether the pathogen origins lie in North America or Eurasia, and (v) determine the source of introduced populations in the Southern Hemisphere.

## 2. Materials and Methods

### 2.1. Sample Collection and Fungal Isolation

In order to cover the largest geographical range possible and to facilitate comparison with previous *D. septosporum* population studies, isolates and/or DNA from earlier studies [20,21,22,23,25,27,28,29,33] were included in this study. Additional needle samples with typical symptoms of DNB were collected from various *Pinus* species across six continents. Many samples were opportunistically collected where infected pine trees were found. Single spore isolations of *D. septosporum* were made using the methods outlined in [34].

Isolates were grouped according to their country of origin and are listed in Table S1. In cases where large unsampled areas occurred between samples, distinct population groups were created to reflect geographical population groups more accurately (e.g., multiple population groups in Canada).

### 2.2. Haplotype and Mating-Type Determination

Isolates were grown in the dark for ca. two weeks at 20 °C on autoclaved cellophane discs (Innovia Films, Wigton, UK) placed on *Dothistroma* medium [35] to obtain mycelium for DNA extraction. DNA was extracted using a Kingfisher Flex magnetic particle processor (Thermo Scientific, Waltham, MA, USA) using Kingfisher Plant DNA extraction kits (Thermo Scientific). Species-specific mating-type primers [36] were used to determine the *Dothistroma* species and mating-type of each isolate as outlined in [27]. Eleven microsatellite markers developed by [37] were used for multilocus haplotyping. Multiplex PCR of the markers (Doth_DS1, Doth_DS2, Doth_E, Doth_F, Doth_G, Doth_I, Doth_J, Doth_K, Doth_L, Doth_M, Doth_O) and fragment analysis were conducted as described by [27].

Isolates with identical multilocus haplotypes (MLHs, i.e., alleles identical at all 11 loci) were considered clones. Two data sets were created: one containing all individuals (non-clone-corrected data set), another containing only one individual of each multilocus haplotype per population (clone-corrected data set).

### 2.3. Genetic Diversity and Differentiation

Five indices were used to evaluate genotypic diversity and were calculated in the R packages poppr [38] and vegan [39] using the non-clone-corrected dataset: (i) Shannon-Wiener index, H [40,41]; (ii) Stoddart and Taylor’s index, G [42]; (iii) Simpson’s index, λ [43]; (iv) genotypic richness, eMLG, the expected number of multilocus genotypes (eMLG) calculated by rarefaction to the smallest sample size (≥10); and (v) genotypic richness, E_5_, an estimation of evenness which is equal to 0 when a single genotype is dominant and increases to 1 as genotypes become more equally represented [41]. The proportion of isolates derived from clones, or asexual reproduction, is known as the clonal fraction (CF) and was calculated according to the method of [44].

The clone-corrected dataset was used to calculate further indices: Nei’s gene diversity, H_exp_ [45], calculated in poppr; the total number of alleles, number of private alleles, and the mean haploid genetic diversity (h) calculated in GENALEX 6.5 [46]; and allelic richness (A_R_) (i.e., the number of distinct alleles in a group) and private allele richness (PA_R_) (i.e., the number of alleles unique to a particular group) calculated in ADZE [47]. The A_R_ and PA_R_ were computed using a rarefaction procedure to adjust them to a specific sample size that allowed comparison between populations having different sample sizes. Calculations were standardized to a uniform size corresponding to the size of the smallest group.

Pairwise *F_ST_* values, used as a measure of population differentiation, and *Nm*, the predicted number of migrants between population groups, were calculated in ARLEQUIN 3.5 [48].

### 2.4. Mating-Type Distribution and Sexual Recombination

An equal proportion of mating-type idiomorphs indicates that sexual reproduction could be frequent enough to maintain equilibrium. To determine whether groups differed significantly from the null hypothesis of a 1:1 ratio of mating-type idiomorphs, an exact binomial test, using two-tailed *p*-values, was used [49].

Poppr [38] was used to calculate the index of association (I_A_) together with its associated measure ($\bar{r_{d}}$). The I_A_ is a measure of multilocus linkage disequilibrium and $\bar{r_{d}}$ is a modification of it that facilitates comparisons between studies by removing the dependency on the number of loci used [50,51]. Sexual populations are expected to have linkage equilibrium due to no linkage among loci, while clonal populations are expected to have significant disequilibrium due to linkage among loci. The I_A_ and $\bar{r_{d}}$ from the observed data were compared to values obtained after 1000 randomizations to simulate random mating.

Both clone-corrected and non-clone-corrected data sets were used for mating tests in order to reduce the chance of rejecting the null hypothesis of random mating that a smaller clone-corrected data set might carry [52].

### 2.5. Population Structure

The population structure of the clone-corrected dataset was assessed using both STRUCTURE and DAPC. STRUCTURE 2.3.4 [53] implements a Bayesian, model-based clustering algorithm to assign individuals to a specified number of clusters (K), minimizing linkage disequilibrium and maximizing Hardy–Weinberg equilibrium within the clusters [54]. To estimate the optimal number of clusters, 60 independent runs of K = 1–15 were carried out in STRUCTURE using no priors (i.e., no information on geographical location or host was provided). Each run had a burn-in of 100,000 iterations followed by 500,000 data-collecting iterations, using a model of correlated allele frequencies and with admixture among populations allowed. CLUMPAK [55] was used to determine the optimal value of K using the ΔK method of [56]. CLUMPAK was used to align all optimum K STRUCTURE runs to the permutation with the highest H-value. The MCL threshold for similarity scores was set to 0.9. The DISTRUCT program [57] was used to visualize the CLUMPP output. Individual haplotypes were assigned to a particular cluster if their membership probability to that cluster was ≥0.8. Additionally, a hierarchical STRUCTURE analysis was done in which the isolates from each of the three major clusters were run in a separate STRUCTURE analysis, with the settings identical to those described above.

To complement the Bayesian approach implemented in STRUCTURE, a multivariate technique that makes no assumptions regarding the population model or data structure was used [58]. Discriminant analysis of principal components (DAPC) was conducted in the R package ADEGENET [58,59]. It is particularly suited to identifying clusters (K) of genetically related individuals as it minimizes variation within groups and maximizes variation between groups [58]. A sequential K-means procedure followed by an assessment of the Bayesian information criterion (BIC) to assess the optimal number of clusters precedes the DAPC analysis itself. Cross-validation was used to determine the optimal number of principal components retained in the analysis [60].

### 2.6. Modeling of Evolutionary History

The STRUCTURE clusters were used to inform and develop historical scenarios describing the evolutionary relationships among populations. These scenarios were investigated using approximate Bayesian computation (ABC) conducted in DIYABC v2.1.0 [61]. The real observed dataset of microsatellite haplotypes is compared with large numbers of simulated datasets (one million per scenario) based on competing for evolutionary scenarios (models). The topology of the scenarios is designed as a composition of events such as separation of one population from another, merging of two populations or change of effective population size. Furthermore, each scenario is characterized by a set of demographical parameters (time of events in the number of generations, effective population size, admixture rate) and a mutational model. Model selection (scenario comparison) is performed via relative posterior probabilities assigned to each scenario resulting from their relative vicinity (of the appropriate simulated datasets) to the observed dataset in a multidimensional space of summary statistics (i.e., usual population genetic characteristics such as gene diversity or Fst which decrease the complexity of the multilocus dataset).

As the number of potential scenarios between a large number of populations is large, cumbersome, and computationally onerous, a stepwise procedure was adopted to build evolutionary scenarios to address specific questions about relationships among two or three STRUCTURE (sub)clusters, where the best scenario in the first step was used to inform the scenarios of the second step (sensu [62]).

The first question about relationships among the North American (NA), Western European (WE) and Eastern European (EE) clusters (K = 3 STRUCTURE result) was assessed via a set of 19 scenarios in ABC analysis 1. The scenarios tested whether all three population clusters were derived separately from an ancestral population or if one cluster was derived from one of the other two, from an unsampled population, or from an admixture event between populations. The dataset contained all samples from Canada and USA, Western and Eastern Europe (243, 546 and 1041 clone-corrected samples for NA, WE and EE, respectively; Table S2).

Subsequently, as the position of the Western European cluster was revealed, the evolutionary relationship between North American and Eastern European clusters was addressed in the fourteen scenarios of ABC analysis 2. These scenarios were based on the premise that one sexual cycle per year was possible and that any genetic exchange between North America and Eurasia was either less than 500 years ago (i.e., the European discovery of America) or 11,000–30,000 years ago (i.e., across the Bering Land Bridge). Tested scenarios included the North American cluster being derived from the Eastern European cluster, and vice versa, either with a bottleneck or without, and either with restrictions on the timing (up to 500 years ago or 11,000–30,000 years ago) or without. Further scenarios included both populations being derived from an unsampled ancestral population, with and without a bottleneck, and with the restrictions on timing described above. Bottleneck events were allowed to range from 0 to 40 generations as continental translocation could be expected to include a longer than usual bottleneck (see Table S2 for a detailed explanation and the historical interpretation of each scenario).

The third question (analysis 3) regarded relationships among the three subclusters of the Eastern European cluster (K = 5 STRUCTURE result), i.e., Northeastern Europe (NEE, 223 samples), Central Europe (CEE, 641 samples) and Turkey (TUR, 82 samples). The same 19 scenarios as in analysis 1 were used.

As the relationship of the Central European subcluster was clarified, analysis 4 centered on determining the relationship between the Northeastern European subcluster and the Turkish subcluster using five scenarios. The scenarios tested whether both populations were derived from an ancestral population independently, if one derived from the other, or from admixture with an unsampled population.

Once the relationship between the three Eastern European subclusters was clarified, the position of the Western European cluster was evaluated in analysis 5 using 11 scenarios. The topology resulting from the previous analyses was retained, and scenarios tested whether the Western European cluster derived from any of the three Eastern European subclusters or an unsampled population or from admixture between any two of these populations.

The final three analyses (6–8) aimed to determine the origins of the introduced populations (with over 10 MLHs) in the Southern Hemisphere. Twenty-two scenarios tested whether the Southern Hemisphere population derived from any of five main populations delimited by STRUCTURE (North America, Western Europe, Central Europe, Northeastern Europe, Turkey) or an unsampled population, or from admixture between any two of these populations. As the Southern Hemisphere populations are known to be recent introductions, the time of their formation was limited to between 1 and 300 generations ago. Three Southern Hemisphere populations were considered—South Africa Hogsback (*n* = 16; analysis 6), South Africa Tzaneen (*n* = 13, analysis 7), and Chile (*n* = 11, analysis 8).

A list and full description of all scenarios is provided in Table S2. Demographic priors of the tested scenarios included effective population size (10 to 10,000), the time of the split or admixture event (in the number of generations ago; 1 to 10,000), the duration of the bottleneck event (in number of generations; 0 to 20), the effective number of founders of a population (2 to 100), and the rate of admixture (0 to 1), except where these differ as specified above. One million datasets were simulated for each scenario. The generalized stepwise model (GSM) was followed for the microsatellite loci, and the default DIYABC values for the priors of the mutation model parameters were used, with the exception of the mean mutation rate, which was extended to a minimum of 1 × 10^−5^. Only classic dinucleotide microsatellite markers fitting the GSM were used in the ABC datasets. Additionally, the highly polymorphic dinucleotide marker L was excluded, resulting in seven markers (DS1, DS2, F, G, I, J, and K) used in the analyses.

For each simulation, a number of commonly used genetic summary statistics (mean number of alleles for one sample and between two samples, mean heterozygosity, F_st_ between two samples, mean index of classification between two samples, and (δµ)^2^ distance between two samples) were used to compare it to the observed dataset using Euclidian distances. The posterior probability of each scenario was then estimated by polychotomous logistic regression on the 1% of simulated datasets closest to the observed dataset [63,64]. Posterior distributions of parameters, model checking using the posterior based error and summary statistics not used in model selection, and confidence in scenario choice using 1000 pseudo-observed test data sets were calculated using the options in DIYABC v2.1.0.

## 3. Results

### 3.1. Isolates and Haplotypes

**Figure 1:**
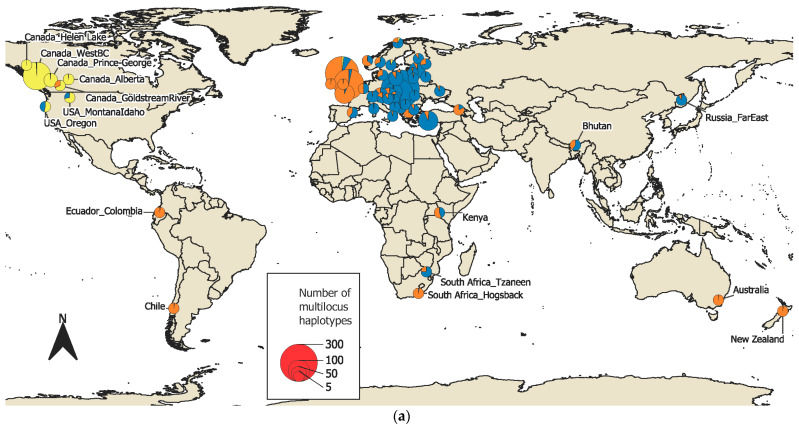

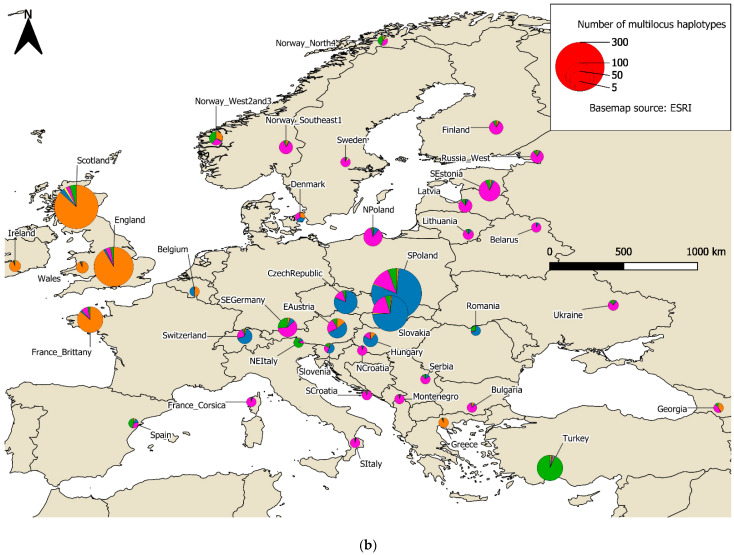
(**a**) World map of *D. septosporum* population groups and their STRUCTURE membership at K = 3; (**b**) European map of *D. septosporum* population groups and their STRUCTURE membership at K = 5.

In total, 3872 *D. septosporum* isolates from 44 countries on six continents were used in this study (Table S1). The isolates were grouped into 56 population groups based on geographical proximity (Figure 1a,b, Table S1). In the vast majority of cases, this corresponded to the county. In some cases, isolates from the same country were placed into separate population groups (e.g., distant geographical groups separated by large areas without isolates in Canada and Norway). Full details of isolates, including the host, geographic location, and population group, are provided in Table S1. Based on the 11 microsatellite markers, these isolates consisted of 1913 unique multilocus haplotypes. All loci were polymorphic with a total of 377 alleles, ranging from 6 at Doth_O to 97 at Doth_L.

### 3.2. Population Structure

**Figure 2:**
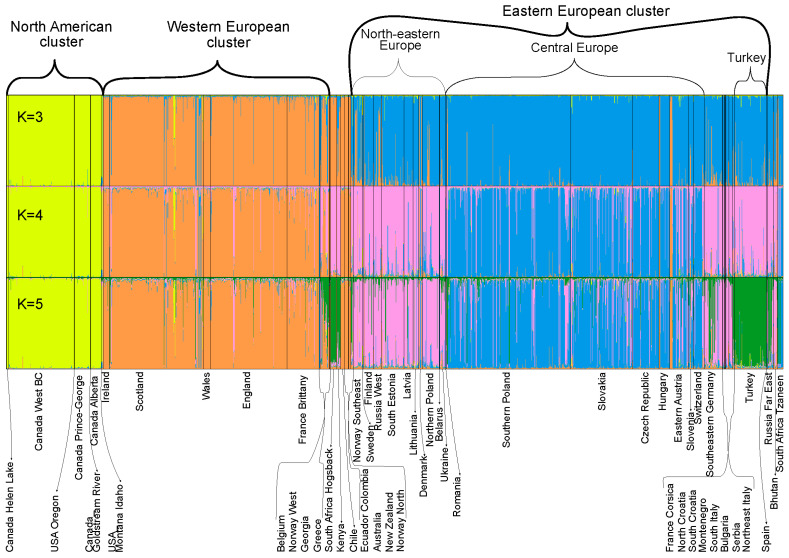
Bayesian clustering of *D. septosporum* multilocus haplotypes inferred using the program STRUCTURE at K = 3, K = 4 and K = 5. Each multilocus haplotype is represented by a vertical line partitioned into colored sections that represent the isolate’s estimated membership fractions in each cluster. Black lines separate isolates from different population groups.

**Figure 3:**
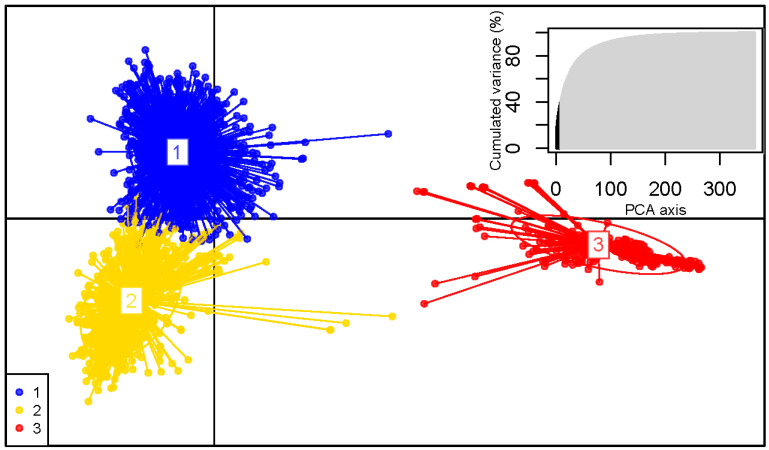
Scatterplot of the discriminant analysis of principal components (DAPC) of *D. septosporum* multilocus haplotypes. Only the first two principal components of the DAPC are displayed. The first axis is the horizontal axis; the second axis is the vertical axis. Group 1 is equivalent to the Eastern European STRUCTURE cluster, group 2 to the Western European STRUCTURE cluster, and group 3 to the North American STRUCTURE cluster. Individual multilocus haplotypes are represented by dots and groups as inertia ellipses. At the top right, the PCA eigenvalues are represented, with the number of principal components used in the optimized analysis in black.

Assessment of the delta K statistic clearly indicated three clusters best explained the data from the STRUCTURE analysis (Figure S1). All 60 independent STRUCTURE runs were concordant (Figure 2 and Figure S2a,b). K-means clustering and inspection of the BIC (Figure S3) from the DAPC analysis also supported three clusters as the best split (Figure 3). The STRUCTURE and DAPC clusters were highly congruent. The clusters were named according to their major geographical occurrences as the North American, Western European and Eastern European clusters. Higher values of K can also be biologically relevant and were examined to discern the substructuring of the populations. At K = 4, a clear subcluster formed of Central European isolates, and at K = 5, the Turkish isolates formed a distinct subcluster (Figure 2). The hieriarchical STRUCTURE analysis, in which each of the three major STRUCTURE clusters was run separately, produced patterns identical to those of the main run at higher values of K. The exception to this was the North American cluster, in which substructuring was seen in a roughly east-west pattern (Figure S4).

### 3.3. Genetic Diversity

Genotypic diversity measures (H, G, λ; Table 1a) showed the lowest diversity in South Croatia and Spain, while the highest was in Southern Poland and Canada West BC. Genetic diversity measures (H_exp_, h, A_R_; Table 1a), on the other hand, showed the highest diversity in Lithuania, Latvia, and South Estonia while the lowest tended to be in South Croatia or in population groups in the Southern Hemisphere such as Australia and New Zealand. While general trends can be seen from these values, they are influenced by the large variation in sample size between individual population groups; therefore, it is more informative to consider the diversity values for the STRUCTURE clusters (K-3) and subclusters (K = 4 and 5). The Eastern European cluster showed the highest level of diversity by almost all measures, both genotypic and genetic (eMLG, H, G, λ, H_exp_, h, A_R_; Table 1b). The Western European and North American clusters had broadly similar levels of diversity. In terms of subclusters, the Central European subcluster had the highest levels of genotypic diversity (eMLG, H, G, lambda) as well as the highest total number of alleles and private alleles, and Turkey had the lowest values for these measures (Table 1c). However, the Central European subcluster also had the greatest number of samples. When rarefied to an equal number of samples (*n* = 82), the Northeastern European subcluster had the highest allelic richness (A_R_), private allele richness (PA_R_), and mean haploid genetic diversity (h) (Table 1c). Values of F_st_ varied greatly and ranged between 0 and 1, with larger values between North American and European population groups and Nm (number of migrants) ranging from 0 to infinity (Table S3).

### 3.4. Mating-Type Distribution and Sexual Recombination

The vast majority of population groups contained both mating-types (Table 2a). The exceptions to this were population groups with a low number of isolates or MLHs (<5) and two Southern Hemisphere population groups (New Zealand, Chile). Sexual recombination is therefore possible in most population groups, and this was supported by the exact binomial test on the mating-type ratios, using both non-clone-corrected and clone-corrected datasets (Table 2a). However, the more sensitive I_A_ and $\bar{r_{d}}$ tests indicate linkage equilibrium, and therefore routine sexual reproduction, in far fewer population groups, particularly using the clone-corrected dataset.

Of the main STRUCTURE clusters, only in the North American cluster was sexual recombination frequently occurring according to the mating-type ratios (Table 2b). The I_A_ and $\bar{r_{d}}$ tests did not support frequent sexual recombination in any of the other STRUCTURE clusters. Regarding the STRUCTURE subclusters, frequent sexual recombination was supported in the Turkish subcluster by both non-clone-corrected and clone-corrected mating-type ratios and in Central Europe by the clone-corrected I_A_ and $\bar{r_{d}}$ tests.

### 3.5. Modeling of Evolutionary History

To determine the evolutionary history of *D. septosporum* from the current set of isolates representing a global collection, 8 sets of scenarios were considered, each addressing a unique question regarding the evolution of *D. septosporum* populations. The STRUCTURE results revealed three main population clusters (North American, Western European and Eastern European) and the first set of ABC scenarios (analysis 1, Table S2) aimed to elucidate the relationship between them. The posterior probabilities were highest for scenarios 1.12 and 1.9 (*p* = 0.4012 and *p* = 0.3821, respectively; Figure S5, Table S2) in which the Western European cluster derived from the Eastern European cluster, either directly (scenario 1.9) or through a weak admixture event with an unsampled population (scenario 1.12), and both the Eastern European and North American clusters derived independently from the ancestral population. Posterior probabilities for scenario 1.9 indicate the Western European population cluster derived from the Eastern European cluster a median of 95.8 and a mode of 45.2 generations ago. For scenario 1.12, an admixture of the Eastern European population cluster and an unsampled population occurred at a median of 46.1 and a mode of 18.7 generations ago (Table S4). Therefore, the posterior probabilities indicate the creation of the Western European cluster occurred around 18–96 generations ago.

The next set of scenarios (analysis 2) was designed to elucidate the relationship between the North American and Eastern European populations. The most supported scenario involved the North American population deriving from an unsampled/ancestral population less than 500 generations ago and the Eastern European population deriving from an unsampled/ancestral population up to 50,000 generations ago (*p* = 0.7593; Table S2). The inverse scenario, with Eastern Europe deriving from an unsampled/ancestral population less than 500 generations ago and the North American population deriving from an unsampled/ancestral population up to 50,000 generations ago was unsupported (*p* = 0.0031), as were scenarios where the North American and Eastern European populations were directly derived from each other or those where the genetic exchange between Eurasia and North America corresponded to the presence of the Bering Land Bridge (*p* < 0.05). Posterior probabilities indicate the North American population derived from the ancestral population a median of 316 and a mode of 434 generations ago with a strong bottleneck (few founders and long duration), whereas the Eastern European population derived from the ancestral population a median of 6460 and a mode of 3210 generations ago (Table S4).

Subsequent sets of scenarios dealt with the relationship between subclusters of the three main STRUCTURE clusters in Eastern Europe. Analysis 3 investigated whether the Central European subcluster, Northeastern European subcluster and Turkish subcluster arose from the ancestral population separately or whether one derived from the other, from an unsampled population, or from admixture, possibly with an unsampled population. This analysis revealed that the Central European subcluster was clearly derived. In the most supported scenario (S3.16, *p* = 0.2218), the Central European subcluster derived from the Northeastern European subcluster. However, confidence intervals overlapped with two other scenarios, which were also well supported (S3.19, *p* = 0.2079 and S3.17, *p* = 0.1957, Figure 4, Table S2). The Central European subcluster was also derived in these scenarios, in the case of S3.19 from admixture between the Northeastern European subcluster and an unsampled population, or in the case of S3.17 from admixture between the Northeastern European subcluster and the Turkish subcluster. Posterior probabilities for the formation of the Central European subcluster were a median of 232 and a mode of 136 generations ago for S3.16, a median of 152 and a mode of 71.2 generations ago for S3.19, and a median of 187 and a mode of 109 generations ago for S3.17. Therefore, the Central European subcluster is likely to have arisen in the range of 70 to 190 generations ago with a weak bottleneck occurring (short duration and a higher number of founders) (Table S4).

The next set of scenarios (analysis 4) considered only Northeastern Europe and Turkey (without the purely derived Central European subcluster) and revealed that the Turkish subcluster was derived from the Northeastern European subcluster (*p* = 0.4116, Figure S5, Table S2). Posterior probabilities indicated the Turkish subcluster arose a median of 985 and a mode of 444 generations ago with a strong bottleneck occurring (long duration and a low number of founders) (Table S4).

**Figure 4:**
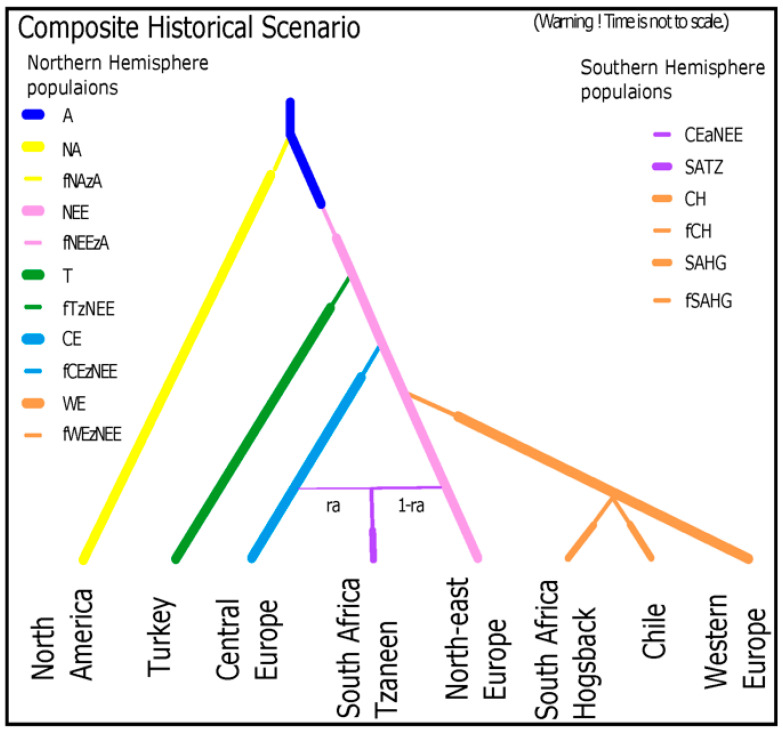
A graphical representation of the overall combined winning historical scenario showing relative timings of cluster and subcluster divergence and colonization events. Founder events and bottlenecks (f in legend) are indicated by thinner lines to indicate a reduction in effective population size. Ra is the rate of admixture and the thickness of lines that indicate the relative contribution from each population. A is the ancestral population, NA the North American cluster, NEE the Northeast European subcluster, T the Turkish subcluster, CE the Central European subcluster, WE the Western European cluster, SATZ the South Africa Tzaneen population group, SAHG the South Africa Hogsback population group, CH the Chile population group.

Analysis 5 revealed that the Western European cluster derived from the Northeastern European subcluster directly and not from the Central European or Turkish subclusters (*p* = 0.4318, Figure S5, Table S2). Posterior probabilities closely corresponded to those in other analyses and demonstrated that the youngest population to diverge was the Western European cluster, followed by the Central European subcluster and finally the Turkish subcluster, which was the oldest population divergence event to take place (Figure 4, Table S4).

Investigation of the origin of the Southern Hemisphere populations revealed that the South Africa Tzaneen population group originated from admixture between the Central European and Northeastern European populations (*p* = 0.4322, Figure S5, Table S2). In contrast, both the South Africa Hogsback and Chile population groups originated from the Western European population (*p* = 0.3602 and *p* = 0.7727, respectively). Other population groups in the Southern Hemisphere contained too few multilocus haplotypes to confidently determine their origin using ABC analyses, yet most of them (Australia, New Zealand, Ecuador and Colombia) belonged strongly to the Western European STRUCTURE cluster, as did Chile, suggesting they too were introduced from the Western European population. Only Kenya differed in its STRUCTURE membership, which is almost identical to the South Africa Hogsback population at all values of K (Figure 2, Figure S2a,b), suggesting a similar origin.

**Figure 5:**
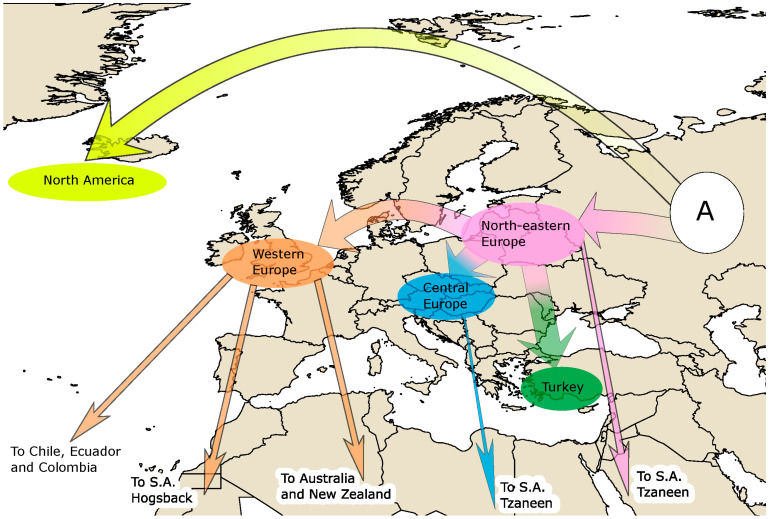
Graphical representation of *D. septosporum* migration pathways (marked by arrows) supported by STRUCTURE and DIYABC analyses. Ancestral population (A) gave rise to both the North American and Eastern European clusters and most likely resided in Eastern Europe and Western Asia. Colors of arrows and ellipses correspond to STRUCTURE cluster colors in Figure 2.

A graphical representation of relative timings of *D. septosporum* population divergence events is given in Figure 4, and a graphical representation of geographical migration events from all historical scenarios is given in Figure 5. Visual inspection of the model checking results confirmed all winning scenarios and their priors fit the real observed dataset well (Figure S6). Confidence in scenario choice for each analysis is given in Table S2, and all posterior distributions of parameters are given in Table S4.

## 4. Discussion

This study constitutes the largest population study ever done on *D. septosporum*, encompassing new and all previously studied populations representing collections from 44 countries, comprising all but three of the countries where the pathogen has been reported from, with over 3800 isolates—with the aim of inferring global evolutionary histories. The population study on this large world-wide collection of *D. septosporum* revealed the presence of three major population clusters (North America, Western Europe and Eastern Europe) and their historical relationships, as well as more fine-scale substructuring of these populations and their interrelations. The North American population cluster was the most genetically distinct and geographically restricted, while the Western European population cluster has spread to much of the Southern Hemisphere. Analysis of historical scenarios crucially revealed that *D. septosporum* is an Old World species being introduced into North America from an ancestral population of Eurasian origin. Historical scenarios also showed many of the European populations ultimately derived from the Northeastern European subcluster indicating that Northeastern Europe and western Asia are likely to be the center of origin of the pathogen. The Turkish subcluster is derived from the Northeastern European subcluster first (i.e., the oldest divergence event), followed by the Central European subcluster and then the Western European cluster (the most recent divergence event).

DNB has been present in North America since at least 1917, when infected needles were collected in Idaho, USA [6,15]. However, dendrochronological studies by [17] suggest that DNB was causing a noticeable growth reduction of *P. contorta* Dougl. ex Loud. var. *latifolia* Engelm. (lodgepole pine) in British Columbia, Canada, as early as the 1830 s. In Europe, on the other hand, DNB was first recorded from needles collected in 1860 in France (as *Hypostomum flichianum* Vuill.), in 1878 in lower Austria (as *Leptostroma pinastri* in de Thümen Mykotheca universalis n° 1278) (Holdenrieder and Queloz, personal communication), in 1880 in Denmark (as *Mycosphaerella pini* Rostr. apud Munk) and in 1910 in European Russia (as *Cytosporina septospora* Dorogin) [6]. The first records in North America and Europe are roughly concurrent; thus, it has long been debated whether *Dothistroma* and *D. septosporum,* in particular, is an Old or New World species. This question is especially relevant as *D. septosporum* was long considered a non-native quarantine organism on the EPPO A2 list of quarantine organisms. However, since the end of 2019, due to its now widespread occurrence in Europe, it has been considered a Regulated Non-Quarantine pest (RNQP) in the European Union.

Historical scenarios developed to elucidate the relationship between North American and Eurasian populations using DIYABC centered around the only known connections between the two continents, i.e., either ca. 11,000 to 30,000 years ago when the Bering Land Bridge allowed human, plant, and animal contact between North America and Asia or ca. 500 years ago till present when European rediscovery of North America again made the transfer of the fungus possible. If the Eurasian population split from an ancestral population less than 500 years ago, or between 11,000 and 30,000 years ago, the ancestral population could have been in the Americas. Conversely, if the North American population split from an ancestral population either less than 500 years ago, or between 11,000 and 30,000 years ago, then the ancestral population could have been in Eurasia. The analysis revealed that the North American population cluster is relatively young and was clearly derived from an ancestral population less than 500 years ago (i.e., ca. 300 generations ago). The Eastern European population cluster was estimated to have derived from the ancestral population in the order of several thousand years ago (i.e., three to seven thousand generations), making a New World ancestral population impossible. The data, therefore, demonstrate that *D. septosporum* is an Old World species. This is supported by the greater genetic and genotypic diversity of the Eastern European population cluster compared to the North American population cluster. Furthermore, all North American isolates formed a distinct, well-supported population cluster in STRUCTURE and DAPC analyses with no further substructuring of the population even at very high values of K (up to K = 15). Conversely, the Eastern European population cluster displayed clear substructuring and high genetic diversity typical of native or long-established populations, whereas no substructuring with lower diversity suggests a derived, or more recently established, population. Only when a hierarchical STRUCTURE analysis was conducted, running the North American isolates independently, were distinct clusters delimited, which resembled those found by [25] (Figure S4). This suggests the structuring of the North American populations is much weaker than that of the European populations.

Nonetheless, the use of a larger number of markers, e.g., SNPs from genotyping-by-sequencing or whole-genome sequencing, and isolates from as yet unsampled regions may reveal new relationships and links. Particular progress could be made elucidating the timing of divergence events. Such progress would help reconcile the findings of Capron et al. (2020), who found Canadian *D. septosporum* populations diverged from each other between 31 and 7 thousand years ago, with the findings of the present study where the North American population diverged more recently.

The distinct Western European population cluster dominates the range from north-western France through the British Isles and Ireland to western Norway. Even though this is a European population, it is clearly differentiated from the Eastern European population cluster, which occurs throughout the rest of Europe and Asia. Modeling of historical scenarios showed that the Western European population arose from the Eastern European population cluster and had no connection with the North American population cluster. Such a connection between Western Europe (i.e., France and Britain) and its ex-colonies in North America would not have been unexpected considering the close trade links and movement of potentially infected material. However, the results using microsatellite markers indicate the Western European cluster derived relatively recently, less than 100 generations ago, and if we consider one generation to occur in one year, then this is after the first evidence of DNB in North America.

Regardless of its recent origin, the Western European population cluster has been spread to much of the Southern Hemisphere where it has caused, and in some countries continues to cause, severe damage to pine plantations [1,9]. Pine species are native to the Northern Hemisphere, with only *P. merkusii*’s native range just crossing the equator [65], and have been introduced to the Southern Hemisphere primarily for timber production [66]. Introductions often followed colonization routes, and afforestation programs began in many areas from the 1870s onward (e.g., Australia, New Zealand, South Africa) [67,68,69]. Introduction of pines was accompanied by or followed by the introduction of *D. septosporum,* and it is noteworthy that many of the Southern Hemisphere countries containing the Western European population cluster were old British colonies (i.e., Australia, New Zealand, South Africa, Kenya), demonstrating a clear route of introduction through increased trade volumes with the colonial power and Western Europe in general. Nonetheless, South American countries that were never British colonies also harbor the Western European population cluster (e.g., Chile, Ecuador and Colombia). Additionally, the Central European and Northeastern European subclusters have been introduced to South Africa Tzaneen, showing that not only the Western European population has been introduced to the Southern Hemisphere. It is clear that there have been at least two to three separate introductions of *D. septosporum* to South Africa as the Hogsback population group originates from the Western European cluster while the Tzaneen population group originates from admixture between the Central European and Northeastern European subclusters.

Although the causal agent of DNB was first clearly described from European Russia in 1911 [13], the disease only achieved notoriety in the 1950s and 1960s due to its severe impact on pine plantations in the Southern Hemisphere, particularly East Africa, New Zealand and Chile [1,8]. Sporadic reports of the disease occurred in Europe until the 1980s, yet without it causing significant damage [1]. It was not until the 1990s that the disease caused severe damage in Europe—in France and Britain [2,70]. The areas where DNB has caused the most damage in both the Southern and Northern Hemispheres are plantations of often exotic species, yet these areas also share the same population cluster of *D. septosporum*—the Western European population cluster. This population is well-differentiated, based on microsatellite markers, from the Eastern European population from which it derived, and differences doubtless occur throughout the genome. It is possible, given the severe damage this population inflicts on its host, that the Western European population cluster has higher virulence compared to the other population clusters. The Western European population cluster is found on 37 host taxa (species, subspecies and varieties) while the Eastern European population cluster has only been found on 23 host taxa and the North American population cluster on only *P. contorta* and its varieties. This increased host range is also suggestive of increased virulence and, coupled with the era of colonization and increased global trade from Western Europe to the Southern Hemisphere, may have contributed to its global success. Variation in virulence levels of the populations should be investigated by rigorous artificial inoculation tests which would also help define the risk further introductions and spread of this population would pose to pine forests worldwide.

At the edge of their distribution ranges, species are known to vary from their core distribution range due to limited gene flow with the core population and increased genetic drift within the edge population, resource limitation, new interspecific interactions, environmental pressures, etc. [71,72,73]. Populations that colonize new habitats beyond their original distribution limits are likely to suffer strong selection pressure due to the intensification of these factors and exposure to new pressures and species competitions [74]. Those populations that survive in new or edge habitats are likely to become locally adapted, with studies of mollusks to mammals and plants showing populations adapted to their local environments [74,75,76], which in some cases are extremely, and unexpectedly, successful when transplanted outside of their local environment [75].

*Dothistroma septosporum* populations are by no means exempt from these pressures and tendencies. Separate populations in new areas are exposed to new host taxa, different foliar microbial communities and competitors, and disparate environmental conditions. This sudden exposure to novel conditions, along with the associated genetic bottleneck, places the fungus under intense pressure to adapt rapidly. A shift to more sexual reproduction in such challenging conditions is likely as sexual reproduction in many ascomycete fungi is associated with adverse conditions [77]. The generation of new genotypes by sexual recombination facilitates survival and adaptation, particularly advantageous in new or changing conditions [78,79]. We hypothesize that the novel host and environmental conditions faced by *D. septosporum* in North America drove the population towards an increased rate of sexual reproduction, as evidenced by the equal proportion of both mating-types, a situation not seen in the Western or Eastern European population clusters. This increased rate of sexual recombination hastened local adaptation and divergence from the Eurasian populations.

This local adaptation may reflect a possible host preference of the North American population group to lodgepole pine, the sole host on which this population cluster was found and a species native to North America. Although the majority of isolates from North America were from lodgepole pine, the two non-lodgepole pine isolates originating from North America did not cluster with the North American population cluster. Additionally, there is strong evidence that the North American population cluster has subsequently been reintroduced into Europe, to Scotland, where it occurs almost exclusively on lodgepole pine [23,80,81]. Such specialization suggests the population cluster could be distinct enough to form a geographical and physiological race, a term used by [80,81] when working with isolates of this population. Once again, artificial inoculation tests, based on the populations presented here, would help clarify not only the virulence but also the degree of host specialization of the major *D. septosporum* global populations.

While the affinity of the North American population group for lodgepole pine is clear, many of the other population clusters and subclusters present a preponderance for certain host taxa. For example, the Western European population cluster is found predominantly on *P. nigra* subsp. *laricio*, *P. sylvestris* and *P. contorta*, the Central European subcluster mainly on *P. nigra* and *P. mugo*, the Northeastern European subcluster on *P. sylvestris* and *P. nigra* and the Turkish subcluster on *P. brutia*. This may indicate some degree of host adaptation, but it is much more likely to be a result of the geographical distribution of the subclusters and the hosts that are grown in these areas, either native species or the non-native species often used in commercial forestry plantations where DNB thrives. Therefore, the presence of *D. septosporum* on particular hosts is typically more influenced by the geographical distribution of the particular *D. septosporum* populations rather than the host specification of the pathogen itself. To what extent specific populations exhibit local adaptation, and whether this is more influenced by host species or environmental conditions (e.g., climate) will require extensive testing under controlled conditions.

The Eastern European population cluster dominates the *D. septosporum* population in much of Europe and Asia from northern Norway and Sweden throughout Europe to Turkey and across to Bhutan and the Russian Far East in Asia. This population cluster has the highest genotypic and genetic diversity of all the population clusters and exhibits significant substructuring. The most prominent subcluster is that of Central Europe, which dominates the Czech Republic, Slovakia, southern Poland, Hungary, Switzerland, Eastern Austria, Slovenia, and Romania. Further subclustering reveals Turkey as a distinct subcluster. Historical scenarios show the Central European subcluster to be derived from the Northeastern European subcluster, although with possible genetic contributions from the Turkish subcluster or an unsampled population. The Central European subcluster was formed relatively recently, ca. 70–190 generations ago, and underwent a weak bottleneck event. In contrast, the Turkish subcluster, clearly derived from the Northeastern European subcluster, was formed long ago, ca. 400–1000 generations ago, and underwent a strong bottleneck event. This weak bottleneck and proximity to Northeastern Europe suggest the Central European subcluster could be derived from the natural spread of the pathogen.

The result that all European population clusters and subclusters (Western European cluster, Central European subcluster, Turkish subcluster) are derived from the Northeastern European subcluster, along with this group’s high genetic diversity strongly suggests the region is part of the center of origin of *D. septosporum* as proposed by [20]. However, the same STRUCTURE population cluster is present in southern, particularly south-eastern, Europe. These population groups were not used for ABC modeling due to the lower numbers of samples from this region. Therefore, this region could encompass the native range of the pathogen as well. This same population cluster is present in Bhutan and the Russian Far East and, although no samples were obtained from much of central Asia, this population could occur throughout the boreal forests of Asia and Europe. The region possesses similar environmental conditions and abundant host species, particularly *P. sylvestris*, whose native range extends across the entire region. *Dothistroma septosporum* produces only mild symptoms on *P. sylvestris* in the region [1,20,82], a situation typical of co-evolved hosts and pathogens [83,84], further supporting the pathogen’s indigenous status in the region.

## Figures and Tables

**Table 1:** Number of *D. septosporum* isolates and summary statistics for (a) the population groups; (b) the three main STRUCTURE clusters; (c) the three Eastern European subclusters.

(**a**)														
**Population** **Group**	**N**	**MLH**	**eMLH ± Standard Error**	**H**	**G**	**λ**	**E_5_**	**H_exp_**	**Clonal Fraction**	**Total Alleles**	**Private Alleles**	**h ± Standard Error**	**AR ± Standard Error**	**PAR ± Standard Error**
**Australia**	4	2	NA	0.562	1.6	0.375	0.795	0.091	0.5	12	0	0.045 ± 0.045	NA	NA
**Belarus**	1	1	NA	NA	NA	NA	NA	NA	NA	NA	NA	NA	NA	NA
**Belgium**	2	2	NA	0.693	2	0.5	1	0.727	0	19	0	0.364 ± 0.07	NA	NA
**Bhutan**	12	11	9.32 ± 0.466	2.369	10.29	0.903	0.958	0.453	0.083	37	1	0.407 ± 0.096	2.357 ± 0.353	0.208 ± 0.112
**Bulgaria**	8	8	NA	2.079	8	0.875	1	0.568	0	34	1	0.497 ± 0.069	2.531 ± 0.265	0.162 ± 0.098
**Canada** **Alberta**	27	26	9.87 ± 0.334	3.244	25.14	0.96	0.979	0.353	0.037	53	14	0.34 ± 0.108	2.11 ± 0.41	0.757 ± 0.392
**Canada Goldstream River**	1	1	NA	NA	NA	NA	NA	NA	NA	NA	NA	NA	NA	NA
**Canada Helen Lake**	4	4	NA	1.386	4	0.75	1	0.394	0	22	0	0.295 ± 0.094	NA	NA
**Canada Prince-George**	55	41	9.38 ± 0.736	3.585	30.56	0.967	0.843	0.518	0.255	70	6	0.506 ± 0.077	2.46 ± 0.325	0.493 ± 0.282
**Canada West BC**	239	168	9.81 ± 0.425	4.98	119.25	0.992	0.818	0.431	0.297	113	26	0.428 ± 0.088	2.215 ± 0.307	0.601 ± 0.239
**Chile**	12	11	9.32 ± 0.466	2.369	10.29	0.903	0.958	0.506	0.083	36	0	0.453 ± 0.08	2.407 ± 0.296	0.099 ± 0.053
**Czech** **Republic**	91	68	9.66 ± 0.561	4.111	53.43	0.981	0.874	0.598	0.253	94	2	0.589 ± 0.071	2.7 ± 0.278	0.146 ± 0.06
**Denmark**	43	10	4.02 ± 1.099	1.454	2.89	0.654	0.577	0.543	0.767	30	0	0.48 ± 0.075	2.366 ± 0.259	0.015 ± 0.013
**Eastern** **Austria**	51	48	9.89 ± 0.316	3.85	45.63	0.978	0.97	0.654	0.059	94	2	0.64 ± 0.062	2.938 ± 0.258	0.119 ± 0.05
**Ecuador and Colombia**	13	9	7.35 ± 0.764	2.032	6.26	0.84	0.794	0.278	0.308	26	0	0.247 ± 0.07	1.459 ± 0.145	0.062 ± 0.062
**England**	596	195	8.76 ± 1.024	4.259	28.96	0.965	0.401	0.554	0.673	96	3	0.551 ± 0.074	2.522 ± 0.236	0.076 ± 0.038
**Finland**	26	24	9.63 ± 0.57	3.131	21.12	0.953	0.919	0.644	0.077	72	2	0.617 ± 0.08	2.935 ± 0.321	0.203 ± 0.071
**France** **Brittany**	282	82	7.55 ± 1.342	3.402	10.27	0.903	0.319	0.584	0.709	72	1	0.576 ± 0.076	2.656 ± 0.251	0.129 ± 0.053
**France** **Corsica**	1	1	NA	NA	NA	NA	NA	NA	NA	NA	NA	NA	NA	NA
**Georgia**	2	1	NA	NA	NA	NA	NA	NA	NA	NA	NA	NA	NA	NA
**Greece**	3	3	NA	1.099	3	0.667	1	0.455	0	19	0	0.303 ± 0.075	NA	NA
**Hungary**	30	27	9.62 ± 0.582	3.245	23.68	0.958	0.92	0.571	0.1	64	0	0.549 ± 0.085	2.672 ± 0.313	0.094 ± 0.049
**Ireland**	20	16	8.72 ± 0.893	2.649	11.76	0.915	0.819	0.552	0.2	36	2	0.515 ± 0.048	2.345 ± 0.151	0.104 ± 0.055
**Kenya**	9	9	NA	2.197	9	0.889	1	0.349	0	22	0	0.31 ± 0.072	1.779 ± 0.218	0.145 ± 0.142
**Latvia**	24	23	9.84 ± 0.369	3.12	22.15	0.955	0.977	0.704	0.042	70	0	0.672 ± 0.074	3.177 ± 0.307	0.144 ± 0.082
**Lithuania**	16	14	9.09 ± 0.714	2.567	11.64	0.914	0.885	0.729	0.125	66	1	0.674 ± 0.065	3.295 ± 0.299	0.219 ± 0.092
**Montenegro**	2	1	NA	NA	NA	NA	NA	NA	NA	NA	NA	NA	NA	NA
**North** **Croatia**	16	3	2.62 ± 0.489	0.777	1.86	0.461	0.727	0.394	0.813	18	0	0.263 ± 0.078	NA	NA
**Northeast Italy**	6	5	NA	1.561	4.5	0.778	0.93	0.346	0.167	21	1	0.274 ± 0.085	1.764 ± 0.254	0.109 ± 0.07
**New Zealand**	24	5	3.07 ± 0.865	0.873	1.71	0.417	0.512	0.164	0.792	16	0	0.131 ± 0.07	1.455 ± 0.247	0.043 ± 0.035
**Norway North**	12	6	5.17 ± 0.665	1.35	2.67	0.625	0.583	0.591	0.5	28	0	0.462 ± 0.075	2.014 ± 0.185	0.054 ± 0.039
**Norway Southeast**	29	23	9.33 ± 0.699	3.08	20.51	0.951	0.939	0.626	0.207	55	1	0.589 ± 0.083	2.855 ± 0.328	0.237 ± 0.12
**Norway West**	52	23	7.41 ± 1.185	2.704	10.65	0.906	0.692	0.613	0.558	44	0	0.581 ± 0.053	2.623 ± 0.201	0.108 ± 0.049
**Northern Poland**	96	44	8.32 ± 1.124	3.336	17.39	0.943	0.605	0.585	0.542	72	2	0.571 ± 0.083	2.704 ± 0.284	0.226 ± 0.084
**Romania**	3	3	NA	1.099	3	0.667	1	0.606	0	23	0	0.404 ± 0.084	NA	NA
**Russia Far East**	18	15	8.97 ± 0.785	2.63	12.46	0.92	0.89	0.521	0.167	40	1	0.48 ± 0.068	2.396 ± 0.263	0.16 ± 0.094
**Russia West**	27	21	8.91 ± 0.896	2.917	15.51	0.936	0.83	0.695	0.222	66	1	0.661 ± 0.064	3.083 ± 0.275	0.123 ± 0.069
**Scotland**	689	240	8.82 ± 1.048	4.502	28.22	0.965	0.305	0.56	0.652	146	22	0.557 ± 0.061	2.505 ± 0.209	0.157 ± 0.099
**South** **Croatia**	8	2	NA	0.377	1.28	0.219	0.612	0.091	0.75	12	1	0.045 ± 0.045	NA	NA
**Southeast Germany**	64	46	9.22 ± 0.836	3.635	28.44	0.965	0.744	0.649	0.281	92	2	0.634 ± 0.079	2.98 ± 0.308	0.333 ± 0.125
**Serbia**	9	9	9 ± 0	2.197	9	0.889	1	0.487	0	31	0	0.428 ± 0.084	2.331 ± 0.278	0.048 ± 0.031
**South** **Estonia**	61	57	9.9 ± 0.306	4.02	53.93	0.981	0.968	0.684	0.066	116	6	0.672 ± 0.082	3.132 ± 0.335	0.218 ± 0.108
**South Italy**	2	1	NA	NA	NA	NA	NA	NA	NA	NA	NA	NA	NA	NA
**Slovakia**	274	157	9.54 ± 0.669	4.725	68	0.985	0.6	0.635	0.427	120	6	0.631 ± 0.068	2.857 ± 0.273	0.103 ± 0.041
**Slovenia**	16	13	8.87 ± 0.743	2.513	11.64	0.914	0.938	0.62	0.188	52	0	0.572 ± 0.075	2.852 ± 0.316	0.17 ± 0.079
**South Africa Hogsback**	20	16	8.72 ± 0.893	2.649	11.76	0.915	0.819	0.378	0.2	26	0	0.354 ± 0.064	1.822 ± 0.153	0.015 ± 0.011
**South Africa Tzaneen**	14	13	9.51 ± 0.5	2.54	12.25	0.918	0.963	0.655	0.071	40	0	0.605 ± 0.051	2.714 ± 0.237	0.018 ± 0.012
**Spain**	13	2	2 ± 0.059	0.54	1.55	0.355	0.768	0.727	0.846	19	0	0.364 ± 0.07	NA	NA
**Southern** **Poland**	602	317	9.7 ± 0.554	5.357	112.06	0.991	0.526	0.618	0.473	115	1	0.616 ± 0.073	2.768 ± 0.27	0.074 ± 0.029
**Sweden**	8	4	NA	1.074	2.29	0.562	0.668	0.727	0.5	31	0	0.54 ± 0.071	NA	NA
**Switzerland**	68	28	6.86 ± 1.333	2.659	7.39	0.865	0.481	0.58	0.588	64	2	0.559 ± 0.068	2.665 ± 0.244	0.245 ± 0.085
**Turkey**	108	82	9.41 ± 0.79	4.181	39.14	0.974	0.592	0.625	0.241	106	9	0.617 ± 0.083	2.871 ± 0.325	0.346 ± 0.093
**Ukraine**	14	14	10 ± NaN	2.639	14	0.929	1	0.622	0	48	0	0.576 ± 0.052	2.713 ± 0.259	0.176 ± 0.117
**USA** **Montana and Idaho**	7	5	NA	1.475	3.77	0.735	0.821	0.718	0.286	35	2	0.575 ± 0.046	3.182 ± 0.263	0.489 ± 0.345
**USA Oregon**	1	1	NA	NA	NA	NA	NA	NA	NA	NA	NA	NA	NA	NA
**Wales**	37	18	6.46 ± 1.293	2.288	5.21	0.808	0.475	0.557	0.514	45	0	0.525 ± 0.063	2.466 ± 0.214	0.036 ± 0.02
**Total**	3872	1913	9.85 ± 0.394	6.864	266.29	0.996	0.277	0.762	0.506	377	NA	NA	NA	NA
(**b**)														
**Cluster**	**N**	**MLH**	**eMLH ± SE**	**H**	**G**	**λ**	**E_5_**	**H_exp_**	**Clonal Fraction**	**Total Alleles**	**Private Alleles**	**h ± SE**	**AR ± Standard Error**	**PAR ± Standard Error**
**Western** **European cluster**	1570	571	190 ± 7.28	5.47	75	0.987	0.312	0.581	0.636	152	38	0.58 ± 0.072	11.026 ± 2.809	3.150 ± 1.721
**Eastern** **European cluster**	1692	1009	268 ± 6.6	6.54	369	0.997	0.531	0.69	0.404	253	112	0.69 ± 0.068	16.265 ± 3.750	6.641 ± 2.524
**North** **American cluster**	331	241	241 ± 0	5.34	171	0.994	0.819	0.549	0.272	157	76	0.547 ± 0.083	14.241 ± 4.045	8.175 ± 3.316
**Total**	3593	1821	267 ± 6.96	6.9	313	0.997	0.315	0.764	0.493	NA	NA	NA	NA	NA
(**c**)														
**Eastern** **European** **Subcluster**	**N**	**MLH**	**eMLH ± SE**	**H**	**G**	**λ**	**E_5_**	**H_exp_**	**Clonal Fraction**	**Total Alleles**	**Private Alleles**	**h ± SE**	**AR ± Standard Error**	**PAR ± Standard Error**
**Central Europe**	1132	653	94.8 ± 3.39	6.11	239.1	0.996	0.53	0.703	0.423	191	44	0.634 ± 0.072	9.677 ± 2.015	1.669 ± 0.595
**Northeastern Europe**	303	225	91.1 ± 3.28	5.18	111.8	0.991	0.624	0.635	0.257	174	35	0.7 ± 0.078	11.947 ± 2.904	3.557 ± 1.575
**Turkey**	108	82	82 ± 0	4.18	39.1	0.974	0.592	0.625	0.241	106	21	0.617 ± 0.083	9.623 ± 1.798	3.062 ± 0.68
**Total**	1543	957	98.8 ± 2.96	6.52	366.9	0.997	0.538	0.692	0.38	NA	NA	NA	NA	NA

N = the number of isolates; MLH = the number of multilocus haplotypes; eMLH = the expected number of multilocus haplotypes, genotypic richness; H = Shannon–Wiener index; G = Stoddart and Taylor’s index; λ = Simpson’s index; E_5_ = estimation of evenness; H_exp_ = Nei’s gene diversity; h = mean haploid genetic diversity; A_R_ = allelic richness; PA_R_ = private allele richness. (a) Diversity indices not calculated for sample sizes less than 3. A_R_ and PA_R_ rarefied to the smallest sample size of 5, populations with less than 5 haplotypes excluded from the calculations. (b) Only individuals with a STRUCTURE membership probability of ≥0.8 to the respective cluster were allocated to the cluster and included in the calculation of diversity indices. A_R_ and PA_R_ were standardized to the smallest group size of 241. (c) The population Central Europe contains population groups Southern Poland, Slovakia, Czech Republic, Hungary, Eastern Austria, Slovenia, Switzerland. The population Northeastern Europe contains population groups Norway Southeast, Sweden, Finland, Russia West, South Estonia, Latvia, Lithuania, Northern Poland, Belarus, Ukraine. Population Turkey contains only Turkey. A_R_ and PA_R_ rarefied to the smallest sample size of 82.

**Table 2:** Mating type ratio and index of association tests for the *D. septosporum* isolates of (a) the population groups; (b) the three main STRUCTURE clusters; (c) the three Eastern European subclusters. Bold *p* values (i.e., those that are non-significant) indicate random mating is supported by the test.

(**a**)												
	**Non-Clone-Corrected**						**Clone-Corrected**					
**Population Group**	***MAT-1-1-1***	***MAT 1-2***	***p* Value**	**I_A_**	$\bar{r_{d}}$	***p* Value** $\left(I_{A}\;\mathrm{and}\;\bar{r_{d}}\right)$	***MAT-1-1-1***	***MAT 1-2***	***p* Value**	**I_A_**	$\bar{r_{d}}$	***p* Value** $\left(I_{A}\;\mathrm{and}\;\bar{r_{d}}\right)$
**Australia**	0	4	NA	NA	NA	NA	0	2	NA	NA	NA	NA
**Belarus**	1	0	NA	NA	NA	NA	1	0	NA	NA	NA	NA
**Belgium**	0	2	NA	NA	NA	NA	0	2	NA	NA	NA	NA
**Bhutan**	5	7	**0.774**	0.154	0.023	**0.288**	5	6	**1**	−0.035	−0.005	**0.502**
**Bulgaria**	2	6	**0.289**	0.897	0.103	0.011	2	6	**0.289**	0.897	0.103	0.009
**Canada Alberta**	13	14	**1**	0.491	0.075	0.009	12	14	**0.845**	0.43	0.067	0.014
**Canada Goldstream River**	0	1	NA	NA	NA	NA	0	1	NA	NA	NA	NA
**Canada Helen Lake**	0	4	NA	NA	NA	NA	0	4	NA	NA	NA	NA
**Canada Prince-George**	20	35	**0.058**	1.67	0.172	<0.001	10	31	0.001	1.55	0.164	<0.001
**Canada West BC**	123	108	**0.357**	0.435	0.05	<0.001	83	77	**0.693**	0.28	0.032	<0.001
**Chile**	0	12	<0.001	0.585	0.067	0.023	0	11	<0.001	0.326	0.037	**0.127**
**Czech Republic**	28	60	<0.001	0.194	0.02	0.002	22	43	0.013	0.083	0.009	**0.154**
**Denmark**	2	41	<0.001	4.3	0.51	<0.001	1	9	0.021	1.27	0.143	0.002
**Eastern Austria**	22	29	**0.401**	0.627	0.064	<0.001	20	28	**0.312**	0.59	0.06	<0.001
**Ecuador and** **Colombia**	1	12	0.003	2.32	0.337	<0.001	1	8	0.039	2.25	0.329	0.002
**England**	217	369	<0.001	0.506	0.054	<0.001	85	100	**0.303**	0.195	0.02	<0.001
**Finland**	14	12	**0.845**	0.82	0.085	<0.001	14	10	**0.541**	0.506	0.052	0.002
**France Brittany**	130	151	**0.233**	1.98	0.213	<0.001	31	50	0.045	0.406	0.043	<0.001
**France Corsica**	0	1	NA	NA	NA	NA	0	1	NA	NA	NA	NA
**Georgia**	2	0	NA	NA	NA	NA	1	0	NA	NA	NA	NA
**Greece**	2	1	NA	NA	NA	NA	2	1	NA	NA	NA	NA
**Hungary**	15	14	1	0.615	0.065	0.002	12	14	**0.845**	0.466	0.05	0.003
**Ireland**	12	8	**0.503**	0.757	0.077	<0.001	8	8	**1.196**	0.468	0.047	0.007
**Kenya**	6	3	**0.508**	0.272	0.039	**0.146**	6	3	**0.508**	0.272	0.039	**0.147**
**Latvia**	10	14	**0.541**	0.45	0.046	**0.32**	10	13	**0.678**	0.406	0.041	**0.474**
**Lithuania**	5	10	**0.302**	0.399	0.042	0.06	5	8	**0.581**	−0.197	−0.021	**0.95**
**Montenegro**	1	1	NA	NA	NA	NA	1	0	NA	NA	NA	NA
**North Croatia**	12	4	**0.077**	3.26	0.664	<0.001	2	1	NA	−0.2	−0.05	**0.632**
**Northeast Italy**	2	4	**0.688**	0.807	0.164	0.023	2	3	1	0.533	0.109	**0.158**
**New Zealand**	0	24	<0.001	0.134	0.071	**0.224**	0	5	**0.063**	−0.455	−0.228	**0.933**
**Norway North**	1	11	0.006	4.17	0.528	<0.001	1	5	**0.219**	3.25	0.411	<0.001
**Norway Southeast**	20	9	**0.061**	1.65	0.189	<0.001	15	8	**0.21**	1.45	0.165	<0.001
**Norway West**	25	27	**0.89**	3.55	0.356	<0.001	10	13	**0.678**	2.14	0.215	<0.001
**Northern Poland**	56	38	**0.079**	1.98	0.211	<0.001	26	16	**0.164**	1.33	0.139	<0.001
**Romania**	3	0	NA	NA	NA	NA	3	0	NA	NA	NA	NA
**Russia Far East**	8	10	**0.815**	0.645	0.072	**0.054**	7	8	**1**	0.477	0.053	**0.236**
**Russia West**	19	7	0.029	1.69	0.171	<0.001	14	6	**0.115**	0.808	0.082	<0.001
**Scotland**	135	546	<0.001	1.67	0.168	<0.001	78	155	<0.001	0.477	0.048	<0.001
**South Croatia**	7	1	**0.07**	NA	NA	NA	1	1	NA	NA	NA	NA
**Southeastern** **Germany**	30	33	**0.801**	0.631	0.067	<0.001	21	24	**0.766**	0.31	0.033	<0.001
**Serbia**	5	4	**1**	0.908	0.115	0.032	5	4	1	0.908	0.115	0.028
**South Estonia**	39	20	0.018	0.466	0.05	**0.129**	38	17	0.006	0.466	0.049	**0.199**
**South Italy**	0	2	NA	NA	NA	NA	0	1	NA	NA	NA	NA
**Slovakia**	133	140	**0.717**	0.313	0.032	<0.001	77	79	**0.936**	0.095	0.01	0.011
**Slovenia**	7	9	**0.804**	0.691	0.08	<0.001	6	7	**1**	0.1	0.012	**0.324**
**South Africa Hogsback**	8	12	**0.503**	0.618	0.071	<0.001	8	8	**1.196**	0.386	0.044	0.009
**South Africa Tzaneen**	8	6	**0.791**	0.903	0.092	<0.001	8	5	**0.581**	0.615	0.063	<0.001
**Spain**	13	0	<0.001	7	1	<0.001	2	0	NA	NA	NA	NA
**Southern Poland**	255	332	0.002	0.512	0.052	<0.001	134	172	0.034	0.327	0.033	<0.001
**Sweden**	6	2	**0.289**	4.72	0.526	<0.001	2	2	NA	−0.545	−0.111	**0.946**
**Switzerland**	39	25	**0.103**	2.77	0.287	<0.001	14	10	**0.541**	1.82	0.185	<0.001
**Turkey**	55	53	**0.923**	0.906	0.104	<0.001	45	37	**0.44**	0.507	0.059	<0.001
**Ukraine**	4	10	**0.18**	−0.011	−0.001	**0.839**	4	10	**0.18**	−0.011	−0.001	**0.833**
**USA Montana and Idaho**	6	1	**0.125**	6.05	0.611	<0.001	4	1	**0.375**	4.5	0.514	<0.001
**USA Oregon**	0	1	NA	NA	NA	NA	0	1	NA	NA	NA	NA
**Wales**	27	9	0.004	1.58	0.165	<0.001	9	8	**1**	−0.027	−0.003	**0.544**
**Total**	1555	2259	<0.001	1.02	0.104	<0.001	868	1057	<0.001	0.969	0.099	<0.001
(**b**)												
	**Non-Clone-Corrected**						**Clone-Corrected**					
**Cluster**	***MAT-1-1-1***	***MAT 1-2***	***p* Value**	**I_A_**	**$\bar{r_{d}}$**	***p* Value** **(** **I_A_** **and** $\bar{r_{d}}$ **)**	***MAT-1-1-1***	***MAT 1-2***	***p* Value**	**I_A_**	$\bar{r_{d}}$	***p*** **Value** **(** **I_A_** **and** $\bar{r_{d}}$ **)**
**Western European cluster**	566	987	<0.001	0.536	0.056	<0.001	220	335	<0.001	0.245	0.025	<0.001
**Eastern European cluster**	767	895	0.002	0.299	0.031	<0.001	459	524	0.041	0.296	0.03	<0.001
**North American cluster**	162	161	**1**	1.193	0.125	<0.001	106	127	**0.19**	1.101	0.115	<0.001
**Total**	1554	2259	<0.001	1.105	0.113	<0.001	828	1031	<0.001	1.014	0.104	<0.001
(**c**)												
	**Non-Clone-Corrected**						**Clone-Corrected**					
**Eastern European Subcluster**	***MAT-1-1-1***	***MAT 1-2***	***p* Value**	**I_A_**	**$\bar{r_{d}}$**	***p* Value** **(** **I_A_** **and** $\bar{r_{d}}$ **)**	***MAT-1-1-1***	***MAT 1-2***	***p* Value**	**I_A_**	**$\bar{r_{d}}$**	***p*** **Value** **(** **I_A_** **and** $\bar{r_{d}}$ **)**
**Central Europe**	499	609	0.001	0.257	0.026	<0.001	279	354	0.003	0.325	0.033	**0.141**
**Northeastern Europe**	175	122	0.002	0.498	0.051	<0.001	129	90	0.01	0.196	0.02	<0.001
**Turkey**	55	53	**0.923**	0.906	0.104	<0.001	45	37	**0.44**	0.507	0.059	<0.001
**Total**	729	784	**0.165**	0.31	0.032	<0.001	453	481	**0.377**	0.298	0.03	<0.001

(b) Only individuals with a STRUCTURE membership probability of ≥0.8 to the respective cluster were allocated to the cluster and included in calculations. (c) Northeastern Europe contains population groups Norway Southeast, Sweden, Finland, Russia West, South Estonia, Latvia, Lithuania, Northern Poland, Belarus, Ukraine. Central Europe contains population groups Southern Poland, Slovakia, Czech Republic, Hungary, Eastern Austria, Slovenia, Switzerland. Population Turkey contains only Turkey.

## Data Availability

The data presented in this study are available in Table S1.

## Supplementary Materials

The following are available online at https://www.mdpi.com/2309-608X/7/2/111/s1, Figure S1: Delta K plot of the STRUCTURE analysis, showing K = 3 as the best clustering of individuals, Figure S2: Bayesian clustering of *D. septosporum* multilocus haplotypes inferred using the program STRUCTURE at K = 6, K = 7 and K = 8 (a) and K = 9, K = 10 and K = 11 (b). Each multilocus haplotype is represented by a vertical line partitioned into colored sections that represent the isolate’s estimated membership fractions in each cluster. Black lines separate isolates from different population groups, Figure S3: Plot of the Bayesian information criterion (BIC) vs. the number of clusters from k-means clustering showing a break at K = 3, indicating three clusters best describe the dataset for DAPC analysis, Figure S4: Bayesian clustering of only the North American *D. septosporum* multilocus haplotypes inferred using the program STRUCTURE at K = 2, K = 3 and K = 4. Each multilocus haplotype is represented by a vertical line partitioned into colored sections that represent the isolate’s estimated membership fractions in each cluster. Black lines separate isolates from different population groups. The inset shows a graph of delta K, Figure S5: Graphical representation of the winning scenarios of demographic history for each of the eight DIYABC analyses. Abbreviations used on time scales refer to time parameters used during simulations, Figure S6: Model checking of the winning DIYABC scenarios for each of the eight conducted analyses, Table S1: Details of *D. septosporum* isolates used in this study including the host, geographic location, population group, microsatellite allele calls and mating-types, Table S2: Scenario descriptions, posterior probabilities with 95% credibility intervals of each scenario, and posterior predictive error of models, Table S3: Pairwise population F_ST_ (below the diagonal) and Nm, the estimated number of migrants per generation (above the diagonal) for the *D. septosporum* population groups, Table S4: Posterior distributions of parameters for winning scenarios of DIYABC analyses 1 to 8.
