# Supplementary material for: Worldwide Genetic Structure Elucidates the Eurasian Origin and Invasion Pathways of Dothistroma septosporum, Causal Agent of Dothistroma Needle Blight
Source: J Fungi (Basel). 2021 Feb 3;7(2):111. doi: 10.3390/jof7020111 (PMC7913368; doi:10.3390/jof7020111)
Supplement: Supplementary file 1 [file jof-07-00111-s001.zip › Sup Table 2 Scenario Descriptions5.pdf]

Supplementary Table 2 Scenario descriptions, posterior probabilities with 95% credibility intervals of each scenario, and posterior predictive error of models.

Question	Scenario	Description of scenarios (the selected scenario is in bold characters)	Posterior probability	95% credibility interval
Analysis 1 – The relationship between the three major STRUCTURE clusters (Western Europe, Eastern Europe, and North America).<br><br>Posterior predictive error (computed over 1000 data sets):<br>Direct approach: 0.651<br>Logistic approach: 0.638	1.1	Western Europe, Eastern Europe, and North America arose independently from an ancestral population	0.0036	[0.0000,0.0260]
	1.2	Eastern Europe arose from an unsampled population which derived from the ancestral population. Western Europe and North America arose independently from the ancestral population.	0.0013	[0.0000,0.0239]
	1.3	Eastern Europe arose from the Western European population. The Western European and North American populations arose independently from the ancestral population.	0.0533	[0.0324,0.0742]
	1.4	Eastern Europe arose from the North American population. The North American and Western Europe arose independently from the ancestral population.	0.0003	[0.0000,0.0230]
	1.5	Eastern Europe arose from admixture between the North American population and the Western European population. The North American and Western European populations arose independently from the ancestral population.	0.0103	[0.0000,0.0324]
	1.6	Eastern Europe arose from admixture between the Western European population and an unsampled population. The North American and Western Europe arose independently from the ancestral population.	0.0545	[0.0336,0.0754]
	1.7	Eastern Europe arose from admixture between the North American population and an unsampled population. The North American, Western European and unsampled populations arose independently from the ancestral population.	0.0004	[0.0000,0.0230]
	1.8	Western Europe arose from an unsampled population which derived from the ancestral population. Eastern Europe and North America arose independently from the ancestral population.	0.0053	[0.0000,0.0276]
	**1.9**	**Western Europe arose from the Eastern European population. The Eastern European and North American populations arose independently from the ancestral population.**	**0.3821**	**[0.3535,0.4107]**
	1.10	Western Europe arose from the North American population. The North American and Eastern Europe arose independently from the ancestral population.	0.0001	[0.0000,0.0228]
	1.11	Western Europe arose from admixture between the North American and Eastern European populations. The North American and Eastern European populations arose independently from the ancestral population.	0.0755	[0.0544,0.0965]
	**1.12**	**Western Europe arose from admixture between the Eastern European population and an unsampled population. The North American and Eastern European populations arose independently from the ancestral population.**	**0.4012**	**[0.3691,0.4332]**
	1.13	Western Europe arose from admixture between the North American population and an unsampled population. The North American and Eastern European populations arose independently from the ancestral population.	0.0018	[0.0000,0.0244]
	1.14	North America arose from an unsampled population which derived from the ancestral population. Eastern Europe and Western Europe arose independently from the ancestral population.	0.0061	[0.0000,0.0284]
	1.15	North America arose from the Eastern European population. The Eastern European and Western European populations arose independently from the ancestral population.	0.0006	[0.0000,0.0233]
	1.16	North America arose from the Western European population. The Western European and Eastern European populations arose independently from the ancestral population.	0.0003	[0.0000,0.0229]
	1.17	North America arose from admixture between the Western European and Eastern European populations. The Western European and Eastern European populations arose independently from the ancestral population.	0.0002	[0.0000,0.0229]

	1.18	North America arose from admixture between the Eastern European population and an unsampled population. The Western European and Eastern European populations arose independently from the ancestral population.	0.0025	[0.0000,0.0251]
	1.19	North America arose from admixture between the Western European population and an unsampled population. The Western European and Eastern European populations arose independently from the ancestral population.	0.0007	[0.0000,0.0234]
Analysis 2 – Is *Dothistroma septosporum* an Old World or New World species?<br><br>Posterior predictive error (computed over 1000 data sets):<br>Direct approach: 0.291<br>Logistic approach: 0.256	2.1	The Eastern European population and the North American population both derived from an unsampled (ancestral) population independently with no bottleneck event. This scenario represents natural spread and change of both populations in situ.	0.0146	[0.0122,0.0170]
	2.2	The Eastern European population derived from the North American population with no bottleneck event 10 to 50,000 generations ago. This represents natural spread and divergence of the North American population into Eurasia.	0.0276	[0.0238,0.0314]
	2.3	The North American population derived from the Eastern European population with no bottleneck event 10 to 50,000 generations ago. This represents natural spread and divergence of the Eastern European population into North American.	0.0283	[0.0245,0.0321]
	2.4	The Eastern European population and the North American population both derived from an unsampled (ancestral) population independently with a bottleneck event. This scenario represents both populations being introduced via a bottleneck event.	0.0171	[0.0143,0.0199]
	2.5	The Eastern European population derived from the North American population between 10 and 50,000 generations ago with a bottleneck event. This represents introduction (via a bottleneck event) directly from Eastern Europe to North America.	0.0255	[0.0220,0.0291]
	2.6	The North American population derived from the Eastern European population between 10 and 50,000 generations ago with a bottleneck event. This represents introduction (via a bottleneck event) directly from North America to Eastern Europe.	0.046	[0.0401,0.0520]
	2.7	An unsampled (ancestral) population occurs in America (Central America or Eastern North America). The North American population derived from an unsampled population between 10 and 50,000 generations ago with no bottleneck event. The Eastern European population derived from the unsampled population between 10 and 500 generations ago with a bottleneck event. This scenario represents founding of the Eastern European population by an unsampled North American population after the discovery of North America by Europeans.	0.0031	[0.0017,0.0046]
	**2.8**	**An unsampled (ancestral) population occurs in Eurasia. The North American population derived from the unsampled population between 10 and 500 generations ago with a bottleneck event. The Eastern European population derived from the unsampled population between 10 and 50,000 generations ago with no bottleneck event. This scenario represents founding of the North American population by an unsampled Eurasian population after the discovery of North America by Europeans.**	**0.7593**	**[0.7438,0.7748]**
	2.9	The Eastern European population derived from the North American population between 10 and 500 generations ago with a bottleneck event. This represents migration directly from Eastern Europe to North America after the discovery of America by Europeans.	0.000	[0.0000,0.0000]
	2.10	The North American population derived from the Eastern European population between 10 and 500 generations ago with a bottleneck event. This represents migration directly from North America to Eastern Europe after the discovery of America by Europeans.	0.005	[0.0037,0.0062]
	2.11	An unsampled (ancestral) population occurs in America (Central America or Eastern North America). The North American population derived from this unsampled population between 10 and 50,000 generations ago without a bottleneck. The Eastern European population derived from the unsampled population between 11,000 and 30,000 generations ago with a bottleneck event. This scenario represents founding of the Eastern European population by an unsampled North American	0.014	[0.0119,0.0161]

		population via the Bering land bridge.		
	2.12	An unsampled (ancestral) population occurs in Eurasia. The North American population derived from the unsampled population between 11,000 and 30,000 generations ago with a bottleneck event. The Eastern European population derived from the unsampled population between 1 and 50,000 generations ago without a bottleneck. This scenario represents founding of the North American population by an unsampled Eurasian population via the Bering land bridge.	0.0116	[0.0097,0.0134]
	2.13	The Eastern European population derived from the North American population between 11,000 and 30,000 generations ago with a bottleneck event. This represents migration directly from Eurasia to North America via the Bering land bridge.	0.0282	[0.0247,0.0317]
	2.14	The North American population derived from the Eastern European population between 11,000 and 30,000 generations ago with a bottleneck event. This represents migration directly from North America to Eurasia via the Bering land bridge.	0.0196	[0.0170,0.0222]
Analysis 3 – The relationship between the three major Eastern European STRUCTURE subclusters (Central Europe, North-eastern Europe, and Turkey).<br><br>Posterior predictive error (computed over 1000 data sets): Direct approach: 0.698 Logistic approach: 0.661	3.1	The North-eastern European, Turkish, and Central European populations arose independently from an ancestral population	0.0114	[0.0000,0.0239]
	3.2	The Turkish population arose from an unsampled population which derived from the ancestral population. The North-eastern European and Central European populations arose independently from the ancestral population.	0.0119	[0.0000,0.0243]
	3.3	The Turkish population arose from the North-eastern European population. The North-eastern European and Central European populations arose independently from the ancestral population.	0.0031	[0.0000,0.0160]
	3.4	The Turkish population arose from the Central European population. The Central European and North-eastern European arose independently from the ancestral population.	0.0082	[0.0000,0.0208]
	3.5	The Turkish population arose from admixture between the Central European population and the North-eastern European population. The Central European and North-eastern European populations arose independently from the ancestral population.	0.006	[0.0000,0.0187]
	3.6	The Turkish population arose from admixture between the North-eastern European population and an unsampled population. The Central European and North-eastern European arose independently from the ancestral population.	0.0058	[0.0000,0.0185]
	3.7	The Turkish population arose from admixture between the Central European population and an unsampled population. The Central European, North-eastern European and unsampled populations arose independently from the ancestral population.	0.0134	[0.0011,0.0257]
	3.8	The North-eastern European population arose from an unsampled population which derived from the ancestral population. The Turkish and Central European populations arose independently from the ancestral population.	0.0041	[0.0000,0.0170]
	3.9	The North-eastern European population arose from the Turkish population. The Turkish population and Central European populations arose independently from the ancestral population.	0.0017	[0.0000,0.0146]
	3.10	The North-eastern European population arose from the Central European population. The Central European and Turkish populations arose independently from the ancestral population.	0.1293	[0.1173,0.1412]
	3.11	The North-eastern European population arose from admixture between the Central European and Turkish populations. The Central European and Turkish populations arose independently from the ancestral population.	0.0263	[0.0146,0.0379]
	3.12	The North-eastern European population arose from admixture between the Turkish population and an unsampled population. The Central European and Turkish populations arose independently from the ancestral population.	0.002	[0.0000,0.0150]
	3.13	The North-eastern European population arose from admixture between the Central European population and an unsampled population. The Central European and Turkish populations arose independently from the ancestral population.	0.1333	[0.1205,0.1461]
	3.14	The Central European population arose from an unsampled population which derived from the ancestral population. The Turkish and North-eastern European populations arose independently from the ancestral population.	0.0073	[0.0000,0.0200]
	3.15	The Central European population arose from the Turkish population. The Turkish and North-eastern European populations arose independently from the ancestral population.	0.0037	[0.0000,0.0165]

	**3.16**	**The Central European population arose from the North-eastern European population. The North-eastern European and Turkish populations arose independently from the ancestral population.**	**0.2218**	**[0.2034,0.2402]**
	3.17	The Central European population arose from admixture between the North-eastern European and Turkish populations. The North-eastern European and Turkish populations arose independently from the ancestral population.	0.1957	[0.1782,0.2132]
	3.18	The Central European population arose from admixture between the Turkish population and an unsampled population. The North-eastern European and Turkish populations arose independently from the ancestral population.	0.0071	[0.0000,0.0198]
	3.19	The Central European population arose from admixture between the North-eastern European population and an unsampled population. The North-eastern European and Turkish populations arose independently from the ancestral population.	0.2079	[0.1906,0.2252]
Analysis 4 – The relationship between the North-eastern European and Turkish subclusters<br><br>Posterior predictive error (computed over 1000 data sets): Direct approach: 0.626 Logistic approach: 0.621	4.1	The North-eastern European and Turkish populations arose independently from an ancestral population.	0.0839	[0.0779,0.0899]
	**4.2**	**The Turkish population arose from the North-eastern European population. The North-eastern European population arose independently from the ancestral population.**	**0.4116**	**[0.4007,0.4224]**
	4.3	The Turkish population arose from admixture between the North-eastern European population and an unsampled population. The North-eastern European population and unsampled populations arose independently from the ancestral population.	0.2672	[0.2572,0.2771]
	4.4	The North-eastern European population arose from the Turkish population. The Turkish population arose independently from the ancestral population.	0.1335	[0.1261,0.1409]
	4.5	The North-eastern European population arose from admixture between the Turkish population and an unsampled population. The Turkish population and the unsampled population arose independently from the ancestral population.	0.1038	[0.0976,0.1101]
Analysis 5 – The relationship of the Western European population to the three Eastern European subpopulations<br><br>Posterior predictive error (computed over 1000 data sets):	ALL	All scenarios in this analysis retain the topology elucidated in the previous analyses. Namely that the North-eastern European population derived from an ancestral population and subsequently the Turkish and Central European populations derived from the North-eastern European population. Only the position of the Western European population changes.		
	5.1	The Western European population derives directly from an ancestral population.	0.0055	[0.0000,0.0139]
	5.2	The Western European population derives from an unsampled population which itself derives from an ancestral population.	0.0079	[0.0000,0.0162]
	5.3	The Western European population derives from an admixture event between the Turkish population and an unsampled population. The unsampled population derives from the ancestral population.	0.0096	[0.0013,0.0179]
	5.4	The Western European population derives from an admixture event between the Central European population and an unsampled population. The unsampled population derives from the ancestral population.	0.0173	[0.0092,0.0254]
	5.5	The Western European population derives from an admixture event between the North-eastern European population and an unsampled population. The unsampled population derives from the ancestral population.	0.2773	[0.2602,0.2945]
	5.6	The Western European population derives from the Turkish population.	0.0041	[0.0000,0.0126]

Direct approach : 0.631<br>Logistic approach: 0.598	5.7	The Western European population derives from an admixture event between the Turkish population and the Central European population.	0.0071	[0.0000,0.0155]
	5.8	The Western European population derives from an admixture event between the Turkish population and the North-eastern European population.	0.1573	[0.1445,0.1701]
	**5.9**	**The Western European population derives from the North-eastern European population.**	**0.4318**	**[0.4137,0.4499]**
	5.10	The Western European population derives from an admixture event between the North-eastern European population and the Central European population.	0.0753	[0.0671,0.0834]
	5.11	The Western European population derives from the Central European population.	0.0068	[0.0000,0.0151]
Analysis 6 – The origin of the South Africa Hogsback population<br><br>Posterior predictive error (computed over 1000 data sets):<br>Direct approach: 0.520<br>Logistic approach: 0.450	ALL	All scenarios in this analysis retain the topology elucidated in the previous analyses. Namely that the North-eastern European population derived from an ancestral population and subsequently the Turkish, Central European, and Western European populations derived from the North-eastern European population. The North American population derived independently from the ancestral population 1 to 500 generations ago.		
	6.1	The South Africa Hogsback population derived from the ancestral population.	0	[0.0000,0.0069]
	6.2	The South Africa Hogsback population derived from the North American population.	0	[0.0000,0.0000]
	**6.3**	**The South Africa Hogsback population derived from the Western European population.**	**0.3602**	**[0.3472,0.3733]**
	6.4	The South Africa Hogsback population derived from the Central European population.	0.0001	[0.0000,0.0070]
	6.5	The South Africa Hogsback population derived from the North-eastern European population.	0.0083	[0.0018,0.0148]
	6.6	The South Africa Hogsback population derived from the Turkish population.	0	[0.0000,0.0069]
	6.7	The South Africa Hogsback population derived from an unsampled population. The unsampled population derived from the ancestral population.	0.0003	[0.0000,0.0071]
	6.8	The South Africa Hogsback population derives from an admixture event between the North American population and the Western European population.	0.0396	[0.0327,0.0465]
	6.9	The South Africa Hogsback population derives from an admixture event between the North American population and the Central European population.	0.0001	[0.0000,0.0069]
	6.10	The South Africa Hogsback population derives from an admixture event between the North American population and the North-eastern European population.	0.0039	[0.0000,0.0106]
	6.11	The South Africa Hogsback population derives from an admixture event between the North American population and the Turkish population.	0	[0.0000,0.0000]
	6.12	The South Africa Hogsback population derives from an admixture event between the North American population and an unsampled population. The unsampled population derived from the ancestral population.	0	[0.0000,0.0069]
	6.13	The South Africa Hogsback population derives from an admixture event between the Western European population and the Central European population.	0.1257	[0.1178,0.1336]
	6.14	The South Africa Hogsback population derives from an admixture event between the Western European population and the North-eastern European population.	0.2838	[0.2703,0.2973]
	6.15	The South Africa Hogsback population derives from an admixture event between the Western European population and the	0.1002	[0.0922,0.1082]

		Turkish population.		
	6.16	The South Africa Hogsback population derives from an admixture event between the Western European population and an unsampled population. The unsampled population derived from the ancestral population.	0.0535	[0.0471,0.0599]
	6.17	The South Africa Hogsback population derives from an admixture event between the Central European population and the North-eastern European population.	0.0057	[0.0000,0.0123]
	6.18	The South Africa Hogsback population derives from an admixture event between the Central European population and the Turkish population.	0.0003	[0.0000,0.0072]
	6.19	The South Africa Hogsback population derives from an admixture event between the Central European population and an unsampled population. The unsampled population derived from the ancestral population.	0.0008	[0.0000,0.0077]
	6.20	The South Africa Hogsback population derives from an admixture event between the North-eastern European population and the Turkish population.	0.0068	[0.0002,0.0134]
	6.21	The South Africa Hogsback population derives from an admixture event between the North-eastern European population and an unsampled population. The unsampled population derived from the ancestral population.	0.0104	[0.0039,0.0169]
	6.22	The South Africa Hogsback population derives from an admixture event between the Turkish population and an unsampled population. The unsampled population derived from the ancestral population.	0.0003	[0.0000,0.0072]
Analysis 7 – The origin of the South Africa Tzaneen population<br><br>Posterior predictive error (computed over 1000 data sets): Direct approach: 0.610 Logistic approach: 0.490	ALL	All scenarios in this analysis retain the topology elucidated in the previous analyses. Namely that the North-eastern European population derived from an ancestral population and subsequently the Turkish, Central European, and Western European populations derived from the North-eastern European population. The North American population derived independently from the ancestral population 1 to 500 generations ago.		
	7.1	The South Africa Tzaneen population derived from the ancestral population.	0	[0.0000,0.0000]
	7.2	The South Africa Tzaneen population derived from the North American population.	0	[0.0000,0.0000]
	7.3	The South Africa Tzaneen population derived from the Western European population.	0.0001	[0.0000,0.0087]
	7.4	The South Africa Tzaneen population derived from the Central European population.	0.1593	[0.1457,0.1729]
	7.5	The South Africa Tzaneen population derived from the North-eastern European population.	0.0157	[0.0073,0.0241]
	7.6	The South Africa Tzaneen population derived from the Turkish population.	0.0003	[0.0000,0.0089]
	7.7	The South Africa Tzaneen population derived from an unsampled population. The unsampled population derived from the ancestral population.	0	[0.0000,0.0086]
	7.8	The South Africa Tzaneen population derives from an admixture event between the North American population and the Western European population.	0	[0.0000,0.0086]
	7.9	The South Africa Tzaneen population derives from an admixture event between the North American population and the Central European population.	0.0016	[0.0000,0.0101]
	7.10	The South Africa Tzaneen population derives from an admixture event between the North American population and the North-eastern European population.	0.0003	[0.0000,0.0089]
	7.11	The South Africa Tzaneen population derives from an admixture event between the North American population and the Turkish population.	0	[0.0000,0.0086]

	7.12	The South Africa Tzaneen population derives from an admixture event between the North American population and an unsampled population. The unsampled population derived from the ancestral population.	0	[0.0000,0.0000]
	7.13	The South Africa Tzaneen population derives from an admixture event between the Western European population and the Central European population.	0.0911	[0.0812,0.1010]
	7.14	The South Africa Tzaneen population derives from an admixture event between the Western European population and the North-eastern European population.	0.0025	[0.0000,0.0110]
	7.15	The South Africa Tzaneen population derives from an admixture event between the Western European population and the Turkish population.	0.0004	[0.0000,0.0090]
	7.16	The South Africa Tzaneen population derives from an admixture event between the Western European population and an unsampled population. The unsampled population derived from the ancestral population.	0	[0.0000,0.0086]
	**7.17**	**The South Africa Tzaneen population derives from an admixture event between the Central European population and the North-eastern European population.**	**0.4322**	**[0.4114,0.4531]**
	7.18	The South Africa Tzaneen population derives from an admixture event between the Central European population and the Turkish population.	0.2336	[0.2136,0.2536]
	7.19	The South Africa Tzaneen population derives from an admixture event between the Central European population and an unsampled population. The unsampled population derived from the ancestral population.	0.0103	[0.0021,0.0185]
	7.20	The South Africa Tzaneen population derives from an admixture event between the North-eastern European population and the Turkish population.	0.0516	[0.0425,0.0607]
	7.21	The South Africa Tzaneen population derives from an admixture event between the North-eastern European population and an unsampled population. The unsampled population derived from the ancestral population.	0.0009	[0.0000,0.0095]
	7.22	The South Africa Tzaneen population derives from an admixture event between the Turkish population and an unsampled population. The unsampled population derived from the ancestral population.	0	[0.0000,0.0086]
Analysis 8 – The origin of the Chile population<br><br>Posterior predictive error (computed over 1000 data sets):<br>Direct approach: 0.460<br>Logistic approach: 0.420	ALL	All scenarios in this analysis retain the topology elucidated in the previous analyses. Namely that the North-eastern European population derived from an ancestral population and subsequently the Turkish, Central European, and Western European populations derived from the North-eastern European population. The North American population derived independently from the ancestral population 1 to 500 generations ago.		
	8.1	The Chile population derived from the ancestral population.	0	[0.0000,0.0000]
	8.2	The Chile population derived from the North American population.	0	[0.0000,0.0000]
	**8.3**	**The Chile population derived from the Western European population.**	**0.7727**	**[0.7620,0.7834]**
	8.4	The Chile population derived from the Central European population.	0	[0.0000,0.0008]
	8.5	The Chile population derived from the North-eastern European population.	0.0003	[0.0000,0.0011]
	8.6	The Chile population derived from the Turkish population.	0	[0.0000,0.0000]
	8.7	The Chile population derived from an unsampled population. The unsampled population derived from the ancestral population.	0	[0.0000,0.0008]
	8.8	The Chile population derives from an admixture event between the North American population and the Western European population.	0.0305	[0.0271,0.0339]

	8.9	The Chile population derives from an admixture event between the North American population and the Central European population.	0	[0.0000,0.0008]
	8.10	The Chile population derives from an admixture event between the North American population and the North-eastern European population.	0	[0.0000,0.0008]
	8.11	The Chile population derives from an admixture event between the North American population and the Turkish population.	0	[0.0000,0.0000]
	8.12	The Chile population derives from an admixture event between the North American population and an unsampled population. The unsampled population derived from the ancestral population.	0	[0.0000,0.0000]
	8.13	The Chile population derives from an admixture event between the Western European population and the Central European population.	0.0587	[0.0540,0.0633]
	8.14	The Chile population derives from an admixture event between the Western European population and the North-eastern European population.	0.0802	[0.0745,0.0859]
	8.15	The Chile population derives from an admixture event between the Western European population and the Turkish population.	0.0288	[0.0262,0.0315]
	8.16	The Chile population derives from an admixture event between the Western European population and an unsampled population. The unsampled population derived from the ancestral population.	0.0285	[0.0259,0.0310]
	8.17	The Chile population derives from an admixture event between the Central European population and the North-eastern European population.	0.0001	[0.0000,0.0009]
	8.18	The Chile population derives from an admixture event between the Central European population and the Turkish population.	0	[0.0000,0.0008]
	8.19	The Chile population derives from an admixture event between the Central European population and an unsampled population. The unsampled population derived from the ancestral population.	0	[0.0000,0.0008]
	8.20	The Chile population derives from an admixture event between the North-eastern European population and the Turkish population.	0.0001	[0.0000,0.0009]
	8.21	The Chile population derives from an admixture event between the North-eastern European population and an unsampled population. The unsampled population derived from the ancestral population.	0	[0.0000,0.0008]
	8.22	The Chile population derives from an admixture event between the Turkish population and an unsampled population. The unsampled population derived from the ancestral population.	0	[0.0000,0.0008]

In the analyses the datasets consisted of populations as follows:
North America (n=243) contained population groups Canada Alberta, Canada Goldstream River, Canada Helen Lake, Canada Prince-George, Canada West BC, USA Montana and Idaho, USA Oregon.
Western Europe (n=546) contained population groups England, France Brittany, Ireland, Norway West, Scotland, Wales.
Eastern Europe (n=1041) contained population groups Belarus, Bulgaria, Czech Republic, Denmark, Eastern Austria, Finland, France Corsica, Hungary, Latvia, Lithuania, Montenegro, North Croatia, Northeast Italy, Norway North, Norway Southeast, Northern Poland,

Romania, Russia West, South Croatia, Southeastern Germany, Serbia, South Estonia, South Italy, Slovakia, Slovenia, Spain, Southern Poland, Sweden, Switzerland, Turkey, Ukraine.

North-eastern Europe (n=223) contained population groups Norway Southeast, Sweden, Finland, Russia West, South Estonia, Latvia, Lithuania, Northern Poland, Belarus, Ukraine.

Central Europe (n=641) contained population groups Southern Poland, Slovakia, Czech Republic, Hungary, Eastern Austria, Slovenia, Switzerland.

Turkey (n=82) contained only the Turkish population group. South Africa Hogsback (n=16), South Africa Tzaneen (n=13), and Chile (n=11) contained only their respective population groups.
