# Supplementary material for: Worldwide Genetic Structure Elucidates the Eurasian Origin and Invasion Pathways of Dothistroma septosporum, Causal Agent of Dothistroma Needle Blight
Source: J Fungi (Basel). 2021 Feb 3;7(2):111. doi: 10.3390/jof7020111 (PMC7913368; doi:10.3390/jof7020111)
Supplement: Supplementary file 1 [file jof-07-00111-s001.zip › Sup Table 4 Posterior Distributions.pdf]

Supplementary Table 4 Posterior distributions of parameters for winning scenarios of DIYABC analyses 1 to 8

Analysis 1 Scenario 9

Parameter	mean	median	mode	q(0.025)	q(0.050)	q(0.250)	q(0.750)	q(0.950)	q(0.975)
NA	3990.0	3660.0	3190.0	1570.0	1850.0	2830.0	4690.0	7810.0	8890.0
WE	5220.0	4980.0	4400.0	1980.0	2330.0	3710.0	6550.0	9000.0	9460.0
EE	8630.0	8870.0	9730.0	6050.0	6540.0	8010.0	9470.0	9900.0	9960.0
A	3920.0	3430.0	935.0	199.0	341.0	1550.0	6000.0	9020.0	9450.0
WEzEE	159.0	95.8	45.2	17.5	23.6	54.7	172.0	493.0	715.0
bWE_EE	10.7	10.9	20.0	0.4	1.5	6.0	15.5	19.3	20.0
fWE_EE	44.7	41.6	17.9	4.3	6.5	21.4	66.2	92.2	96.0
NAzA	4110.0	3670.0	285.0	137.0	255.0	1400.0	6560.0	9270.0	9610.0
bNA_A	12.2	13.2	20.0	1.3	2.1	8.0	17.2	19.6	20.0
fNA_A	43.2	39.2	6.4	3.6	5.2	19.1	65.8	92.4	95.7
EEzA	4590.0	4250.0	2350.0	756.0	1010.0	2440.0	6520.0	9200.0	9600.0
bEE_A	8.5	7.9	0.0	0.0	0.5	3.4	13.2	18.4	19.0
fEE_A	49.3	48.4	30.8	4.6	7.0	25.4	73.3	94.1	96.8
µmic_1	0.0	0.0	0.0	0.0	0.0	0.0	0.0	0.0	0.0
pmic_1	0.3	0.3	0.3	0.2	0.2	0.3	0.3	0.3	0.3

snimic_1	0.0	0.0	0.0	0.0	0.0	0.0	0.0	0.0	0.0
pmic_1	0.3	0.3	0.3	0.2	0.2	0.3	0.3	0.3	0.3
snimic_1	0.0	0.0	0.0	0.0	0.0	0.0	0.0	0.0	0.0

Analysis 1 Scenario 12

Parameter	mean	median	mode	q(0.025)	q(0.050)	q(0.250)	q(0.750)	q(0.950)	q(0.975)
NA	3440.0	3110.0	2780.0	1300.0	1540.0	2380.0	4040.0	6830.0	8210.0
WE	5480.0	5290.0	4090.0	1940.0	2310.0	3860.0	6980.0	9210.0	9600.0
EE	8150.0	8360.0	9860.0	5200.0	5670.0	7280.0	9230.0	9860.0	9920.0
A	4430.0	4100.0	1970.0	271.0	475.0	2060.0	6680.0	9230.0	9640.0
U	4680.0	4470.0	484.0	236.0	458.0	2190.0	7110.0	9370.0	9680.0
UaEE	81.5	46.1	18.7	4.4	6.5	22.2	93.1	263.0	370.0
bUaEE	9.6	9.3	5.2	0.1	1.1	5.0	14.2	18.7	19.6
fUaEE	47.5	44.9	23.5	5.4	8.3	25.0	69.0	93.4	96.4
ra	0.8	0.9	1.0	0.3	0.4	0.8	0.9	1.0	1.0
EEzA	4160.0	3690.0	1660.0	519.0	757.0	2000.0	6030.0	8950.0	9430.0
bEE_A	8.3	7.6	0.0	0.0	0.5	3.2	13.0	18.3	18.9
fEE_A	56.8	59.4	92.7	6.1	9.9	34.5	80.7	96.3	98.1

NAzA	3910.0	3400.0	149.0	126.0	233.0	1300.0	6190.0	9100.0	9540.0
bNA_A	9.5	9.3	0.7	0.5	0.8	4.6	14.4	18.7	20.0
fNA_A	42.5	38.6	2.4	3.3	4.8	18.2	65.0	91.8	95.8
UzA	1050.0	458.0	105.0	30.6	51.0	184.0	1160.0	4370.0	6220.0
bU	10.4	10.6	19.2	0.0	1.2	5.4	15.5	19.2	20.0
fU	47.5	45.6	3.6	3.8	5.8	22.7	71.4	93.8	97.1
µmic_1	0.0	0.0	0.0	0.0	0.0	0.0	0.0	0.0	0.0
pmic_1	0.3	0.3	0.3	0.2	0.2	0.3	0.3	0.3	0.3
snimic_1	0.0	0.0	0.0	0.0	0.0	0.0	0.0	0.0	0.0

Analysis 2 Scenario 8

Parameter	mean	median	mode	q(0.025)	q(0.050)	q(0.250)	q(0.750)	q(0.950)	q(0.975)
NA	6380.0	6480.0	8680.0	2220.0	2690.0	4640.0	8260.0	9660.0	9830.0
EE	8150.0	8410.0	9820.0	5050.0	5600.0	7300.0	9240.0	9860.0	9930.0
A	4550.0	4180.0	1400.0	343.0	565.0	2020.0	6970.0	9370.0	9680.0
txNAzA	308.0	316.0	434.0	86.2	110.0	213.0	409.0	482.0	491.0
bxNAzA	23.4	24.5	30.1	3.0	5.1	15.6	32.1	38.3	39.2
fNAzA	36.3	30.7	15.1	4.2	6.1	16.7	51.6	85.0	91.3

tEEzA	9400.0	6460.0	3210.0	1190.0	1580.0	3660.0	11700.0	28700.0	36900.0
μmic_1	0.0	0.0	0.0	0.0	0.0	0.0	0.0	0.0	0.0
pmic_1	0.3	0.3	0.3	0.2	0.2	0.3	0.3	0.3	0.3
snimic_1	0.0	0.0	0.0	0.0	0.0	0.0	0.0	0.0	0.0

Analysis 3 Scenario 16

Parameter	mean	median	mode	q(0.025)	q(0.050)	q(0.250)	q(0.750)	q(0.950)	q(0.975)
CE	8420.0	8640.0	9550.0	5590.0	6130.0	7720.0	9380.0	9880.0	9940.0
NEE	8310.0	8510.0	8920.0	5530.0	6050.0	7560.0	9260.0	9840.0	9920.0
TK	5140.0	4830.0	4120.0	2040.0	2370.0	3640.0	6370.0	9060.0	9530.0
A	2820.0	2040.0	257.0	127.0	197.0	847.0	4240.0	8070.0	8980.0
CEzNEE	328.0	232.0	136.0	55.1	71.2	143.0	383.0	892.0	1210.0
bCE_NEE	5.0	3.5	0.0	0.0	0.0	1.2	7.5	15.1	17.4
fCE_NEE	56.1	57.6	75.6	7.0	11.2	34.4	78.7	95.7	97.8
NEEzA	2920.0	2260.0	1150.0	407.0	552.0	1280.0	3940.0	7780.0	8740.0
bNEE_A	7.9	7.1	0.0	0.1	0.4	3.0	12.3	17.8	18.6
fNEE_A	58.7	61.9	98.9	6.1	10.0	36.6	82.9	97.0	98.6
TKzA	1940.0	1170.0	345.0	68.1	121.0	482.0	2700.0	6460.0	7890.0
bTK_A	9.2	8.8	0.7	0.0	0.7	4.1	14.1	18.6	19.2

fTK_A	51.4	51.7	68.1	4.7	7.3	27.0	75.5	95.3	97.8
µmic_1	0.0	0.0	0.0	0.0	0.0	0.0	0.0	0.0	0.0
pmic_1	0.3	0.3	0.3	0.2	0.2	0.2	0.3	0.3	0.3
snimic_1	0.0	0.0	0.0	0.0	0.0	0.0	0.0	0.0	0.0

Analysis 3 Scenario 19

Parameter	mean	median	mode	q(0.025)	q(0.050)	q(0.250)	q(0.750)	q(0.950)	q(0.975)
CE	7860.0	8160.0	9630.0	4190.0	4860.0	6870.0	9130.0	9830.0	9910.0
NEE	8470.0	8670.0	9690.0	5810.0	6340.0	7790.0	9350.0	9870.0	9940.0
TK	5900.0	5740.0	5570.0	2600.0	3020.0	4510.0	7200.0	9270.0	9620.0
A	2340.0	1510.0	226.0	85.9	139.0	609.0	3310.0	7500.0	8620.0
U	5000.0	4990.0	4060.0	342.0	661.0	2580.0	7400.0	9480.0	9740.0
UaNEE	234.0	152.0	71.2	17.9	25.6	79.6	283.0	714.0	926.0
bUaNEE	4.1	2.7	0.0	0.0	0.0	0.8	5.9	13.6	16.3
fUaNEE	57.6	59.3	93.8	8.1	12.9	36.7	79.9	95.9	97.8
ra	0.8	0.8	0.9	0.3	0.4	0.7	0.9	1.0	1.0
NEEzA	3050.0	2390.0	1370.0	366.0	518.0	1340.0	4190.0	7900.0	8750.0
bNEE_A	10.7	11.0	19.4	0.0	1.4	5.9	15.8	19.3	19.7

fNEE_A	52.9	53.6	88.3	5.0	7.6	29.1	77.1	95.2	97.7
TKzA	1730.0	1000.0	296.0	53.9	98.0	431.0	2280.0	6140.0	7610.0
bTK_A	11.6	12.1	20.0	0.9	1.8	6.9	16.6	19.5	20.0
fTK_A	49.7	48.8	33.5	4.5	6.8	25.6	73.4	94.6	97.1
UzA	1560.0	904.0	338.0	241.0	271.0	494.0	1880.0	5430.0	7060.0
bU	7.8	6.8	0.0	0.0	0.3	2.9	12.3	17.9	20.0
fU	53.9	54.3	81.8	5.4	8.4	30.5	78.7	96.2	98.2
μmic_1	0.0	0.0	0.0	0.0	0.0	0.0	0.0	0.0	0.0
pmic_1	0.3	0.3	0.3	0.2	0.2	0.3	0.3	0.3	0.3
snimic_1	0.0	0.0	0.0	0.0	0.0	0.0	0.0	0.0	0.0

Analysis 3 Scenario 17

Parameter	mean	median	mode	q(0.025)	q(0.050)	q(0.250)	q(0.750)	q(0.950)	q(0.975)
CE	7980.0	8260.0	9850.0	4550.0	5170.0	7060.0	9180.0	9840.0	9910.0
NEE	8100.0	8320.0	9890.0	5050.0	5580.0	7230.0	9180.0	9840.0	9920.0
TK	4780.0	4520.0	3980.0	1980.0	2290.0	3480.0	5780.0	8360.0	9100.0
A	3590.0	2960.0	430.0	166.0	280.0	1270.0	5510.0	8790.0	9370.0
NEEaTK	271.0	187.0	109.0	34.0	45.6	110.0	323.0	761.0	1020.0

bNEEaTK	4.1	2.7	0.0	0.0	0.0	0.9	5.9	13.1	15.8
fNEEaTK	57.6	59.3	72.9	9.2	14.0	37.1	79.5	95.7	97.8
ra	0.8	0.8	0.9	0.3	0.5	0.8	0.9	1.0	1.0
NEEzA	3850.0	3350.0	2300.0	563.0	761.0	1930.0	5390.0	8620.0	9240.0
bNEE_A	10.0	9.9	3.8	0.5	1.1	4.9	15.1	19.0	19.6
fNEE_A	55.5	57.1	99.3	5.3	8.9	32.5	79.7	96.3	98.3
TKzA	2760.0	2040.0	832.0	276.0	381.0	1040.0	3870.0	7700.0	8800.0
bTK_A	12.5	13.6	20.0	1.5	2.4	8.3	17.4	19.7	20.0
fTK_A	49.5	49.4	7.9	4.1	6.3	24.3	74.2	94.3	97.3
μmic_1	0.0	0.0	0.0	0.0	0.0	0.0	0.0	0.0	0.0
pmic_1	0.3	0.3	0.3	0.2	0.2	0.2	0.3	0.3	0.3
snimic_1	0.0	0.0	0.0	0.0	0.0	0.0	0.0	0.0	0.0

Analysis 4 Scenario 2

Parameter	mean	median	mode	q(0.025)	q(0.050)	q(0.250)	q(0.750)	q(0.950)	q(0.975)
NEE	8000.0	8280.0	9670.0	4620.0	5140.0	7040.0	9220.0	9850.0	9930.0
TK	6280.0	6230.0	6320.0	3060.0	3480.0	5020.0	7530.0	9290.0	9620.0
A	5090.0	5080.0	5320.0	342.0	630.0	2640.0	7500.0	9510.0	9750.0

TKzNEE	1390.0	985.0	444.0	215.0	271.0	573.0	1750.0	3990.0	5000.0
bTK_NEE	12.6	13.5	20.0	1.6	2.6	8.5	17.2	19.6	20.0
fTK_NEE	33.6	26.3	4.9	3.1	4.1	12.5	50.1	86.1	92.4
NEEzA	5890.0	5950.0	5090.0	1450.0	1910.0	3980.0	7910.0	9530.0	9770.0
bNEE_A	9.5	9.3	0.8	0.0	0.8	4.5	14.4	18.8	20.0
fNEE_A	56.0	58.4	96.2	5.6	9.1	32.5	80.6	96.5	98.3
μmic_1	0.0	0.0	0.0	0.0	0.0	0.0	0.0	0.0	0.0
pmic_1	0.3	0.3	0.3	0.1	0.2	0.2	0.3	0.3	0.3
snimic_1	0.0	0.0	0.0	0.0	0.0	0.0	0.0	0.0	0.0

Analysis 5 Scenario 9

Parameter	mean	median	mode	q(0.025)	q(0.050)	q(0.250)	q(0.750)	q(0.950)	q(0.975)
WE	6440.0	6430.0	6500.0	2840.0	3330.0	5050.0	7890.0	9500.0	9740.0
CE	7940.0	8140.0	8890.0	4750.0	5350.0	7030.0	9060.0	9810.0	9910.0
NEE	7500.0	7670.0	8440.0	4040.0	4570.0	6400.0	8790.0	9730.0	9850.0
TK	4800.0	4480.0	4160.0	1830.0	2160.0	3430.0	5890.0	8640.0	9260.0
A	4670.0	4460.0	593.0	280.0	481.0	2150.0	7130.0	9400.0	9680.0
WEzNEE	140.0	82.5	44.5	14.7	20.1	46.9	154.0	407.0	575.0

bWEzNEE	10.8	11.1	20.0	0.0	1.4	6.2	15.6	19.3	20.0
fWEzNEE	44.6	41.4	24.0	4.9	7.2	21.9	64.9	91.9	95.4
CEzNEE	232.0	145.0	83.2	29.1	36.8	83.0	262.0	722.0	1000.0
bCEzNEE	7.1	6.0	0.0	0.0	0.1	2.5	11.0	17.4	18.8
fCEzNEE	56.5	58.2	85.7	7.2	11.3	34.6	79.6	95.9	97.9
TKzNEE	1220.0	869.0	388.0	160.0	211.0	484.0	1550.0	3500.0	4420.0
bTKzNEE	10.4	10.6	18.4	0.5	1.2	5.6	15.4	19.1	20.0
fTKzNEE	40.8	35.6	2.0	3.3	4.7	16.9	62.4	91.1	95.2
NEEzA	4770.0	4420.0	2680.0	1170.0	1450.0	2790.0	6560.0	9150.0	9600.0
bNEEzA	9.4	9.2	2.3	0.4	0.8	4.4	14.4	18.6	20.0
fNEEzA	46.6	44.6	2.9	3.6	5.4	21.5	70.6	94.0	96.9
μmic_1	0.0	0.0	0.0	0.0	0.0	0.0	0.0	0.0	0.0
pmic_1	0.3	0.3	0.3	0.2	0.2	0.3	0.3	0.3	0.3
snimic_1	0.0	0.0	0.0	0.0	0.0	0.0	0.0	0.0	0.0

Analysis 6 Scenario 3

Parameter	mean	median	mode	q(0.025)	q(0.050)	q(0.250)	q(0.750)	q(0.950)	q(0.975)
SAHG	2170.0	1200.0	159.0	78.0	110.0	423.0	3100.0	7620.0	8650.0

NA	8170.0	8520.0	9980.0	4640.0	5260.0	7260.0	9400.0	9910.0	9960.0
WE	6950.0	7030.0	7410.0	3650.0	4180.0	5900.0	8110.0	9490.0	9730.0
CE	8560.0	8750.0	9310.0	6050.0	6610.0	7980.0	9370.0	9870.0	9940.0
NEE	8490.0	8680.0	8900.0	5940.0	6510.0	7910.0	9260.0	9830.0	9910.0
TK	4910.0	4660.0	4340.0	1850.0	2190.0	3520.0	6040.0	8650.0	9220.0
A	3680.0	3020.0	999.0	264.0	409.0	1390.0	5600.0	8910.0	9440.0
SAHGzWE	99.3	78.8	27.1	10.1	14.2	39.6	145.0	251.0	274.0
bSAHGzWE	13.1	13.9	20.0	2.7	4.1	9.7	17.3	19.7	20.0
fSHzWE	27.3	20.4	12.2	3.4	4.7	11.7	36.1	76.4	86.7
WEzNEE	282.0	171.0	123.0	39.8	50.1	103.0	309.0	861.0	1220.0
bWEzNEE	11.9	12.6	20.0	0.7	2.0	7.5	16.7	19.6	20.0
fWEzNEE	57.4	59.4	100.0	7.3	11.1	35.1	81.1	96.7	98.6
CEzNEE	301.0	188.0	82.9	37.8	50.0	109.0	338.0	893.0	1300.0
bCEzNEE	7.0	5.8	0.0	0.0	0.1	2.2	10.9	17.4	18.8
fCEzNEE	60.4	63.2	95.5	8.1	12.9	40.1	83.0	96.7	98.5
TKzNEE	1260.0	895.0	572.0	168.0	221.0	514.0	1590.0	3590.0	4580.0
bTKzNEE	12.7	13.8	20.0	0.8	2.4	8.7	17.5	19.8	20.0
fTKzNEE	45.7	43.0	11.6	4.0	6.2	21.3	68.6	92.9	96.1

NEEzA	4580.0	4190.0	2860.0	1110.0	1390.0	2660.0	6300.0	8960.0	9480.0
bNEEzA	12.0	12.9	20.0	1.1	2.0	7.7	16.9	19.6	20.0
fNEEzA	49.6	49.0	4.4	4.2	6.2	23.8	74.8	95.1	97.8
NAzA	347.0	366.0	483.0	114.0	144.0	264.0	444.0	490.0	495.0
bNAzA	24.6	26.2	40.0	3.4	5.8	16.9	33.4	38.8	39.6
fNAzA	37.5	31.6	15.6	4.7	6.7	17.7	53.1	87.1	93.0
μmic_1	0.0	0.0	0.0	0.0	0.0	0.0	0.0	0.0	0.0
pmic_1	0.3	0.3	0.3	0.2	0.2	0.3	0.3	0.3	0.3
snimic_1	0.0	0.0	0.0	0.0	0.0	0.0	0.0	0.0	0.0

Analysis 7 Scenario 17

Parameter	mean	median	mode	q(0.025)	q(0.050)	q(0.250)	q(0.750)	q(0.950)	q(0.975)
SATZ	4710.0	4440.0	2010.0	555.0	807.0	2310.0	6970.0	9390.0	9650.0
NA	7640.0	7960.0	9940.0	3690.0	4330.0	6470.0	9110.0	9850.0	9930.0
WE	6490.0	6510.0	6530.0	3010.0	3510.0	5240.0	7790.0	9430.0	9690.0
CE	8750.0	8950.0	9500.0	6450.0	6970.0	8260.0	9460.0	9880.0	9940.0
NEE	7930.0	8090.0	8380.0	4970.0	5540.0	7160.0	8890.0	9720.0	9850.0
TK	5010.0	4760.0	3880.0	1760.0	2120.0	3540.0	6270.0	8880.0	9420.0

A	3280.0	2560.0	588.0	234.0	360.0	1150.0	4880.0	8560.0	9270.0
CEaNEE	64.2	39.7	6.1	3.1	4.5	16.7	88.2	214.0	251.0
bCEaNEE	7.0	6.2	0.0	0.0	0.2	2.9	10.5	16.5	18.3
fCEaNEE	54.9	54.8	52.9	8.8	13.8	35.1	75.6	94.3	96.9
ra	0.6	0.7	0.6	0.2	0.3	0.5	0.8	0.9	0.9
WEzNEE	216.0	129.0	67.4	25.3	33.5	74.0	234.0	658.0	929.0
bWEzNEE	10.4	10.4	20.0	0.4	1.3	5.6	15.3	19.1	20.0
fWEzNEE	53.1	52.9	38.3	6.0	9.7	30.2	76.4	95.4	97.8
CEzNEE	247.0	156.0	92.1	39.0	48.7	95.3	272.0	717.0	1040.0
bCEzNEE	9.4	9.0	4.9	0.0	0.7	4.5	14.1	18.9	19.7
fCEzNEE	60.5	63.7	92.0	8.5	13.4	39.9	83.3	96.9	98.5
TKzNEE	1300.0	933.0	582.0	175.0	236.0	538.0	1610.0	3680.0	4720.0
bTKzNEE	12.8	13.9	20.0	0.0	2.4	8.6	17.6	19.8	20.0
fTKzNEE	45.2	41.9	8.3	4.1	6.1	21.3	68.2	92.9	96.8
NEEzA	4920.0	4610.0	2760.0	1240.0	1550.0	2960.0	6690.0	9180.0	9550.0
bNEEzA	9.9	9.8	3.8	0.4	0.9	5.0	14.9	18.9	20.0
fNEEzA	45.0	42.0	2.0	3.3	4.7	19.7	69.4	93.2	96.5
NAzA	336.0	353.0	495.0	109.0	136.0	245.0	437.0	489.0	495.0

bNAzA	20.2	20.1	21.3	1.8	3.1	11.4	29.2	37.4	38.6
fNAzA	44.5	41.0	25.7	5.9	8.6	23.7	63.6	91.0	95.3
μmic_1	0.0	0.0	0.0	0.0	0.0	0.0	0.0	0.0	0.0
pmic_1	0.3	0.3	0.3	0.2	0.3	0.3	0.3	0.3	0.3
snimic_1	0.0	0.0	0.0	0.0	0.0	0.0	0.0	0.0	0.0

Analysis 8 Scenario 3

Parameter	mean	median	mode	q(0.025)	q(0.050)	q(0.250)	q(0.750)	q(0.950)	q(0.975)
CH	4680.0	4430.0	1080.0	395.0	595.0	2190.0	7140.0	9350.0	9680.0
NA	7830.0	8170.0	9750.0	3990.0	4660.0	6760.0	9230.0	9860.0	9930.0
WE	8670.0	8840.0	9240.0	6410.0	6880.0	8190.0	9350.0	9840.0	9920.0
CE	8320.0	8520.0	9320.0	5490.0	6070.0	7600.0	9260.0	9840.0	9920.0
NEE	8110.0	8310.0	8650.0	5240.0	5750.0	7400.0	9030.0	9770.0	9880.0
TK	4930.0	4710.0	4340.0	1830.0	2220.0	3570.0	6040.0	8600.0	9220.0
A	3410.0	2700.0	886.0	256.0	388.0	1260.0	5160.0	8690.0	9340.0
SHzWE	72.3	48.6	8.8	4.6	6.7	21.5	102.0	225.0	255.0
bCHzWE	9.5	9.1	7.8	0.0	1.1	4.9	13.9	18.6	19.5
fCHzWE	62.8	65.5	96.7	12.2	18.3	45.1	82.9	96.8	98.5

WEzNEE	112.0	66.0	40.1	15.0	18.7	39.9	115.0	327.0	492.0
bWEzNEE	12.8	13.8	20.0	1.5	2.8	8.8	17.5	19.8	20.0
fWEzNEE	43.3	39.3	16.0	4.1	6.0	19.8	64.6	92.2	96.0
CEzNEE	191.0	116.0	63.5	24.7	31.8	68.8	208.0	553.0	811.0
bCEzNEE	7.0	5.8	0.0	0.0	0.1	2.3	10.8	17.3	18.8
fCEzNEE	57.1	59.0	91.3	6.8	11.3	35.7	80.5	96.1	98.1
TKzNEE	1380.0	988.0	521.0	182.0	245.0	563.0	1770.0	3920.0	4870.0
bTKzNEE	10.9	11.3	18.8	0.4	1.4	6.2	16.0	19.3	20.0
fTKzNEE	45.0	41.8	10.4	3.9	5.6	20.2	68.3	92.9	96.2
NEEzA	4140.0	3640.0	1710.0	879.0	1090.0	2180.0	5770.0	8840.0	9390.0
bNEEzA	9.4	9.2	0.0	0.0	0.7	4.3	14.4	18.7	20.0
fNEEzA	46.5	44.4	3.1	3.5	5.2	21.4	70.7	94.0	96.8
NAzA	341.0	360.0	499.0	107.0	137.0	251.0	442.0	490.0	495.0
bNAzA	25.6	27.5	40.0	3.9	6.4	18.3	34.1	39.0	39.6
fNAzA	35.1	28.8	9.4	4.0	5.8	15.3	50.5	85.1	91.2
μmic_1	0.0	0.0	0.0	0.0	0.0	0.0	0.0	0.0	0.0
pmic_1	0.3	0.3	0.3	0.2	0.2	0.3	0.3	0.3	0.3
snimic_1	0.0	0.0	0.0	0.0	0.0	0.0	0.0	0.0	0.0
