# Supplementary material for: Worldwide Genetic Structure Elucidates the Eurasian Origin and Invasion Pathways of Dothistroma septosporum, Causal Agent of Dothistroma Needle Blight
Source: J Fungi (Basel). 2021 Feb 3;7(2):111. doi: 10.3390/jof7020111 (PMC7913368; doi:10.3390/jof7020111)
Supplement: Supplementary file 1 [file jof-07-00111-s001.zip › SupFig5 part2 A4toA8.pdf]

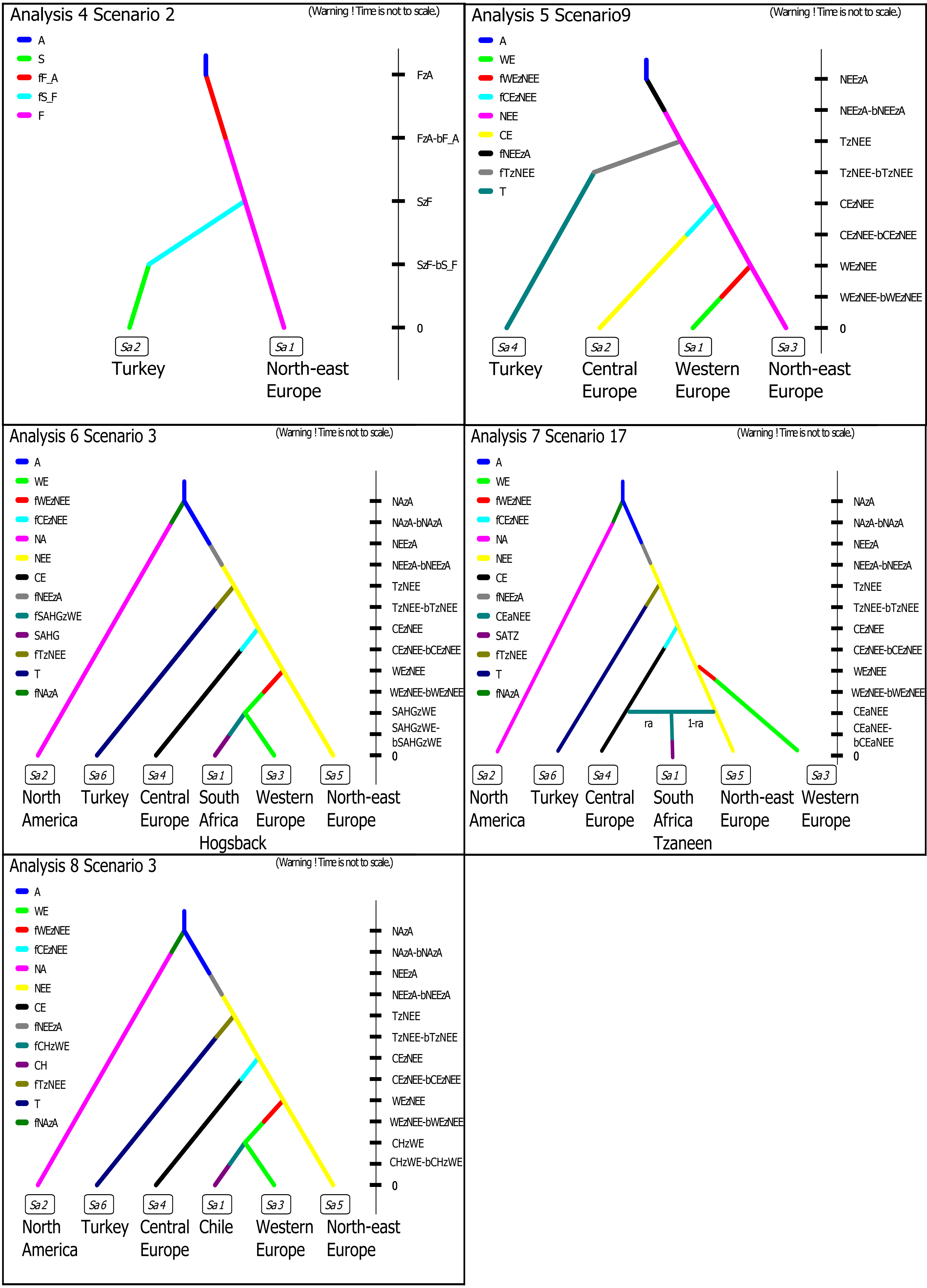

Analysis 4 Scenario 2
(Warning ! Time is not to scale.)
A
S
fF_A
fS_F
F
FzA
FzA-bF_A
SzF
SzF-bS_F
0
Sa 2
Turkey
Sa 1
North-east Europe
Analysis 5 Scenario9
(Warning ! Time is not to scale.)
A
WE
fWEzNEE
fCEzNEE
NEE
CE
fNEEzA
fTzNEE
T
NEEzA
NEEzA-bNEEzA
TzNEE
TzNEE-bTzNEE
CEzNEE
CEzNEE-bCEzNEE
WEzNEE
WEzNEE-bWEzNEE
0
Sa 4
Turkey
Sa 2
Central Europe
Sa 1
Western Europe
Sa 3
North-east Europe
Analysis 6 Scenario 3
(Warning ! Time is not to scale.)
A
WE
fWEzNEE
fCEzNEE
NA
NEE
CE
fNEEzA
fSAHGzWE
SAHG
fTzNEE
T
fNAzA
NAzA
NAzA-bNAzA
NEEzA
NEEzA-bNEEzA
TzNEE
TzNEE-bTzNEE
CEzNEE
CEzNEE-bCEzNEE
WEzNEE
WEzNEE-bWEzNEE
SAHGzWE
SAHGzWE-bSAHGzWE
0
Sa 2
North America
Sa 6
Turkey
Sa 4
Central Europe
Sa 1
South Africa Hogsback
Sa 3
Western Europe
Sa 5
North-east Europe
Analysis 7 Scenario 17
(Warning ! Time is not to scale.)
A
WE
fWEzNEE
fCEzNEE
NA
NEE
CE
fNEEzA
CEaNEE
SATZ
fTzNEE
T
fNAzA
NAzA
NAzA-bNAzA
NEEzA
NEEzA-bNEEzA
TzNEE
TzNEE-bTzNEE
CEzNEE
CEzNEE-bCEzNEE
WEzNEE
WEzNEE-bWEzNEE
CEaNEE
CEaNEE-bCEaNEE
0
ra
1-ra
Sa 2
North America
Sa 6
Turkey
Sa 4
Central Europe
Sa 1
South Africa Tzaneen
Sa 5
North-east Europe
Sa 3
Western Europe
Analysis 8 Scenario 3
(Warning ! Time is not to scale.)
A
WE
fWEzNEE
fCEzNEE
NA
NEE
CE
fNEEzA
fCHzWE
CH
fTzNEE
T
fNAzA
NAzA
NAzA-bNAzA
NEEzA
NEEzA-bNEEzA
TzNEE
TzNEE-bTzNEE
CEzNEE
CEzNEE-bCEzNEE
WEzNEE
WEzNEE-bWEzNEE
CHzWE
CHzWE-bCHzWE
0
Sa 2
North America
Sa 6
Turkey
Sa 4
Central Europe
Sa 1
Chile
Sa 3
Western Europe
Sa 5
North-east Europe
