# Supplementary material for: Worldwide Genetic Structure Elucidates the Eurasian Origin and Invasion Pathways of Dothistroma septosporum, Causal Agent of Dothistroma Needle Blight
Source: J Fungi (Basel). 2021 Feb 3;7(2):111. doi: 10.3390/jof7020111 (PMC7913368; doi:10.3390/jof7020111)

Supplementary Figure 6 Model checking of scenarios

a)Analysis 1 Scenario 12

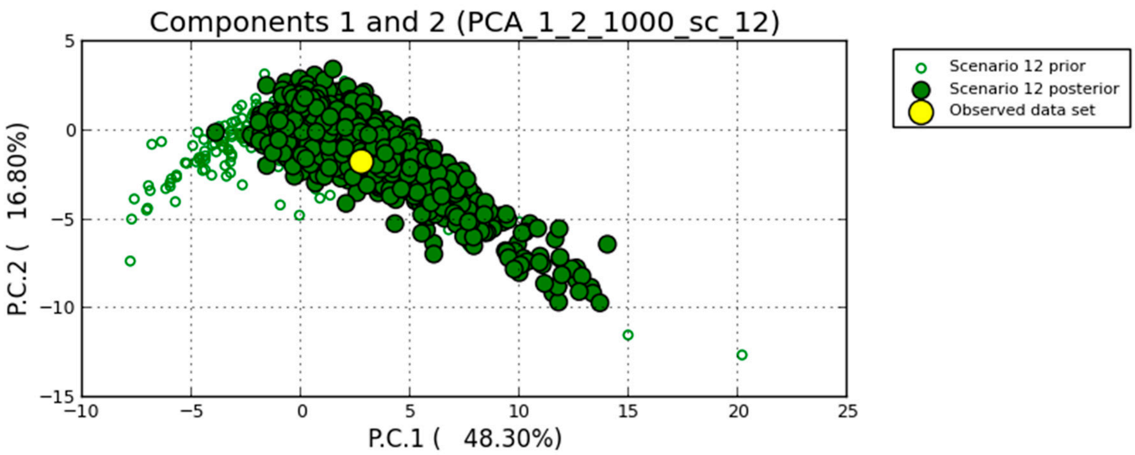

b)Analysis 1 Scenario 9

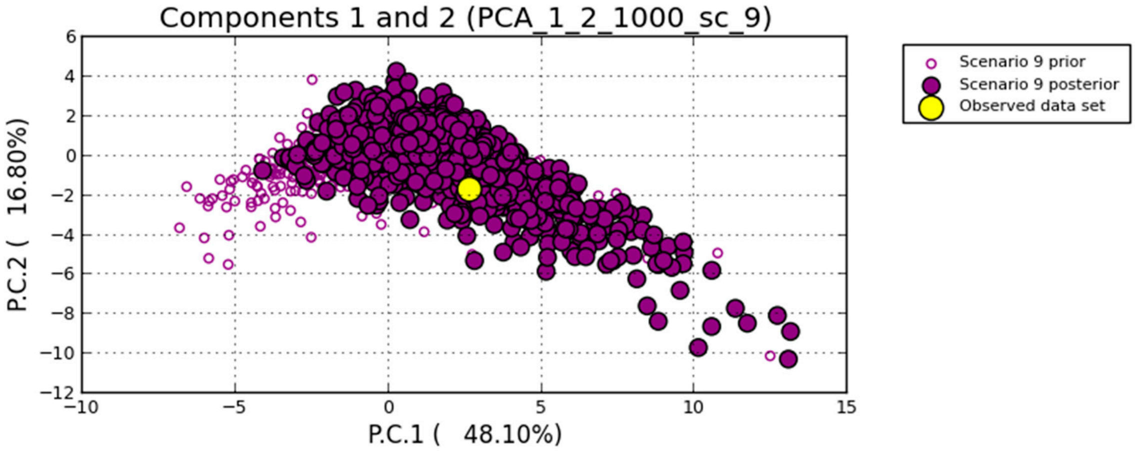

c)Analysis 2 Scenario 4

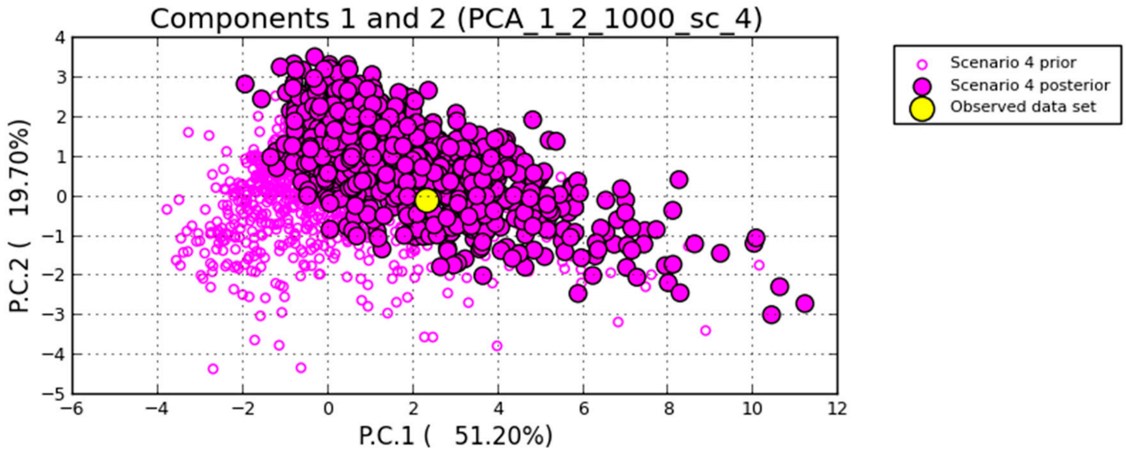

d)Analysis 3 Scenario 16

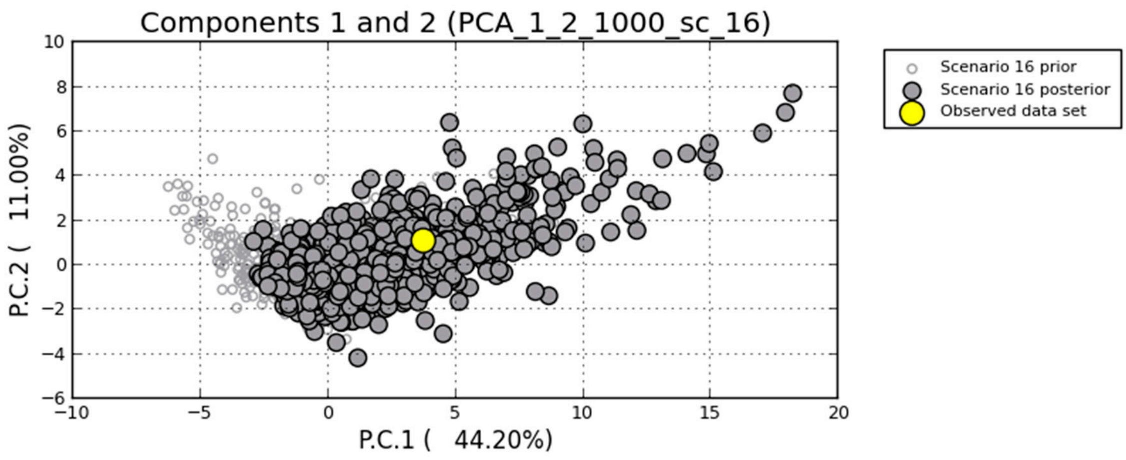

f)Analysis 4 Scenario 2

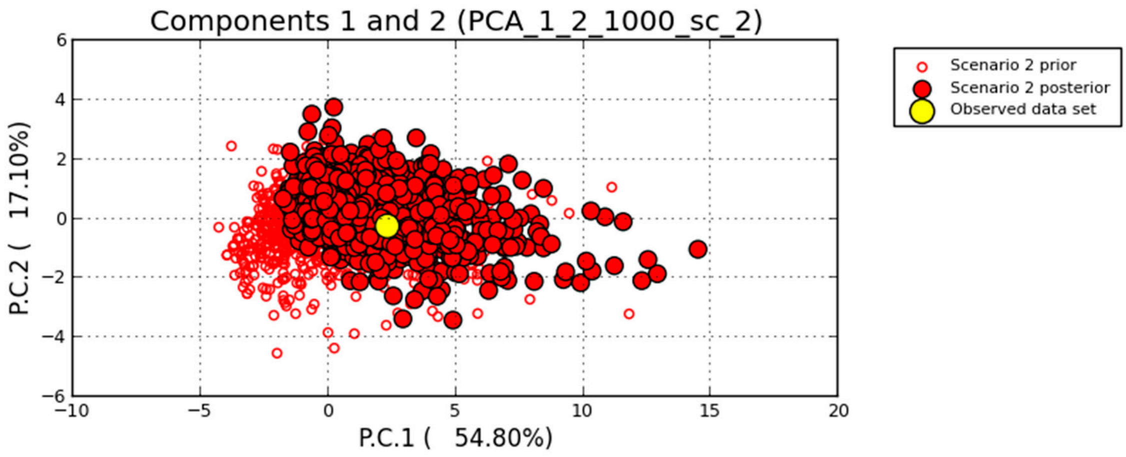

g)Analysis 5 Scenario 9

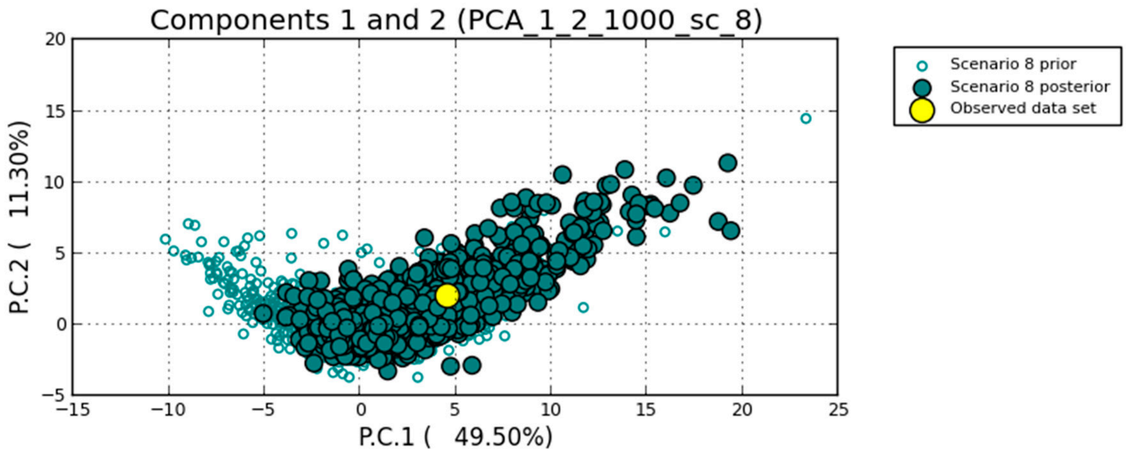

h)Analysis 6 Scenario 3

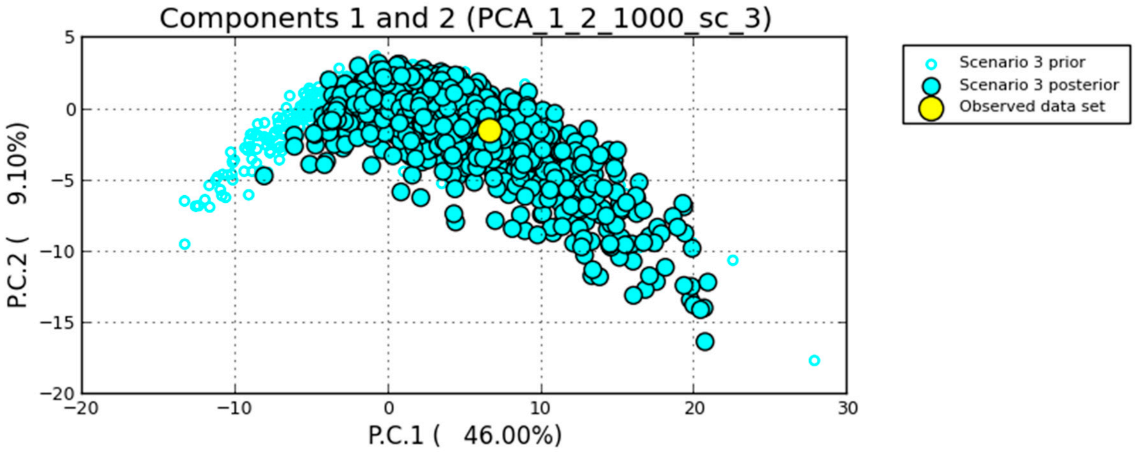

i)Analysis 7 Scenario 17

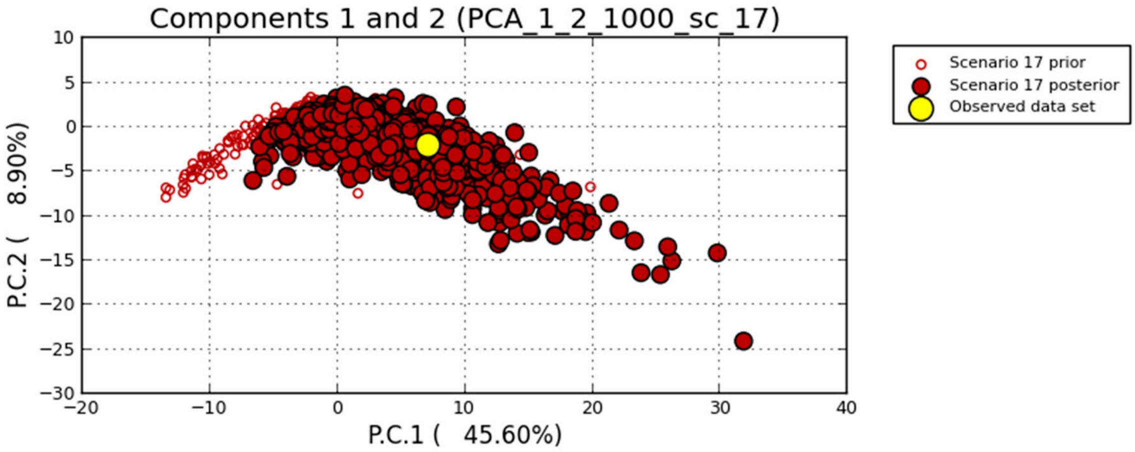

j)Analysis 8 Scenario 3

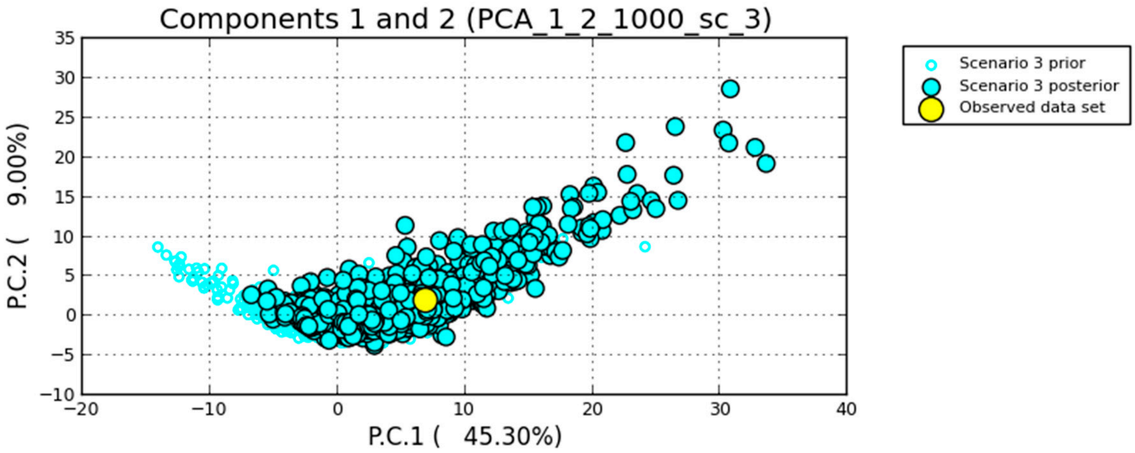

Supplement: Supplementary file 1 [file jof-07-00111-s001.zip › SupFig6 Model checking of scenarios.pdf]
