# Supplementary figures and images for: Worldwide Genetic Structure Elucidates the Eurasian Origin and Invasion Pathways of Dothistroma septosporum, Causal Agent of Dothistroma Needle Blight
Source: J Fungi (Basel). 2021 Feb 3;7(2):111. doi: 10.3390/jof7020111 (PMC7913368; doi:10.3390/jof7020111)

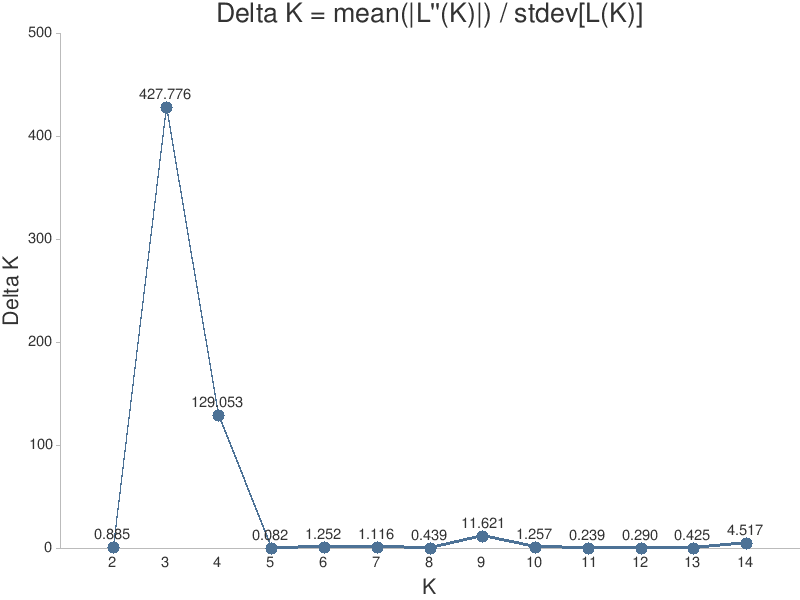

Supplement: Supplementary file 1 [file jof-07-00111-s001.zip › SupFig1_Best_K_By_Evanno-DeltaKByKGraph.png]

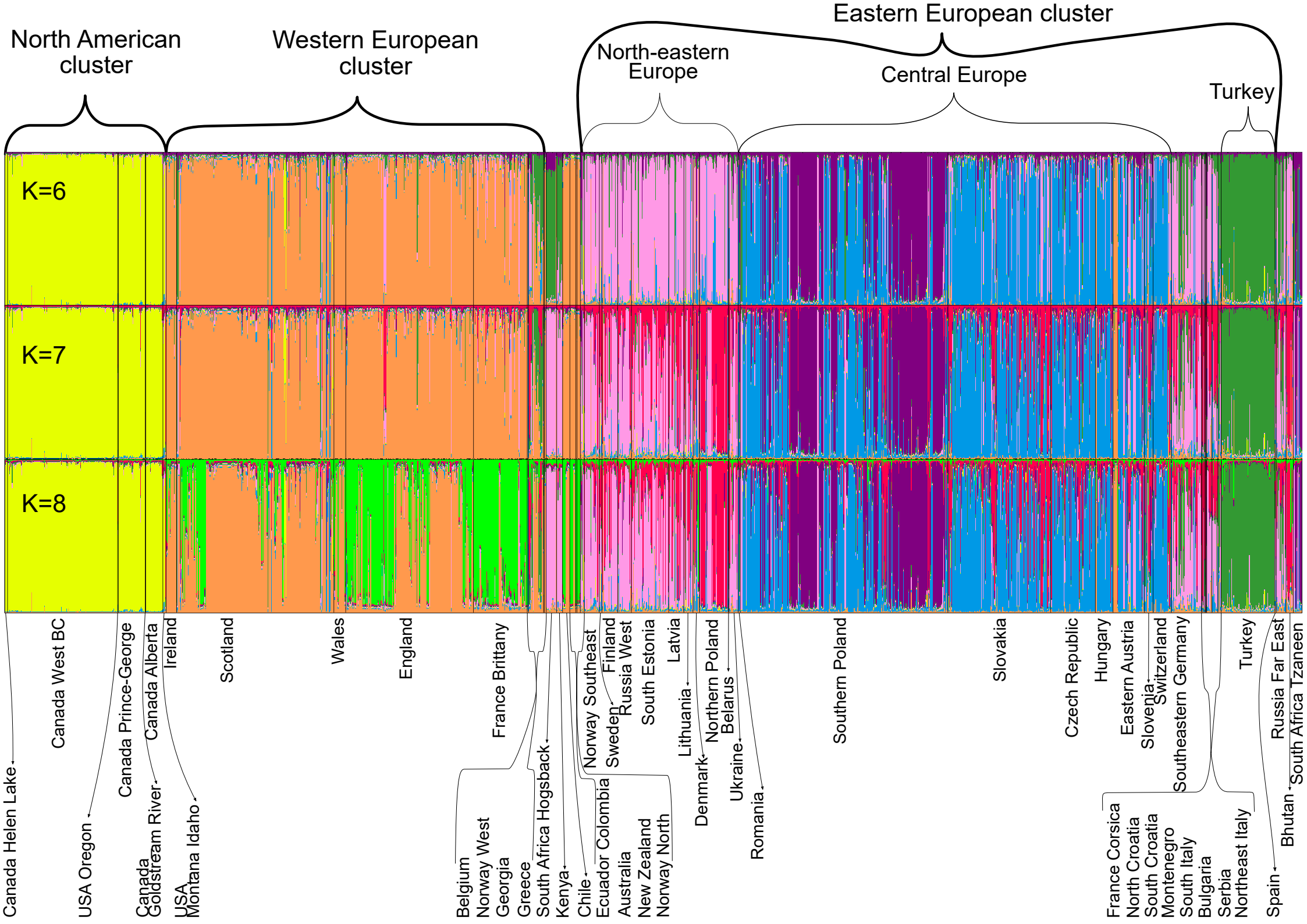

Supplement: Supplementary file 1 [file jof-07-00111-s001.zip › SupFig2aStructureK6to8.pdf]

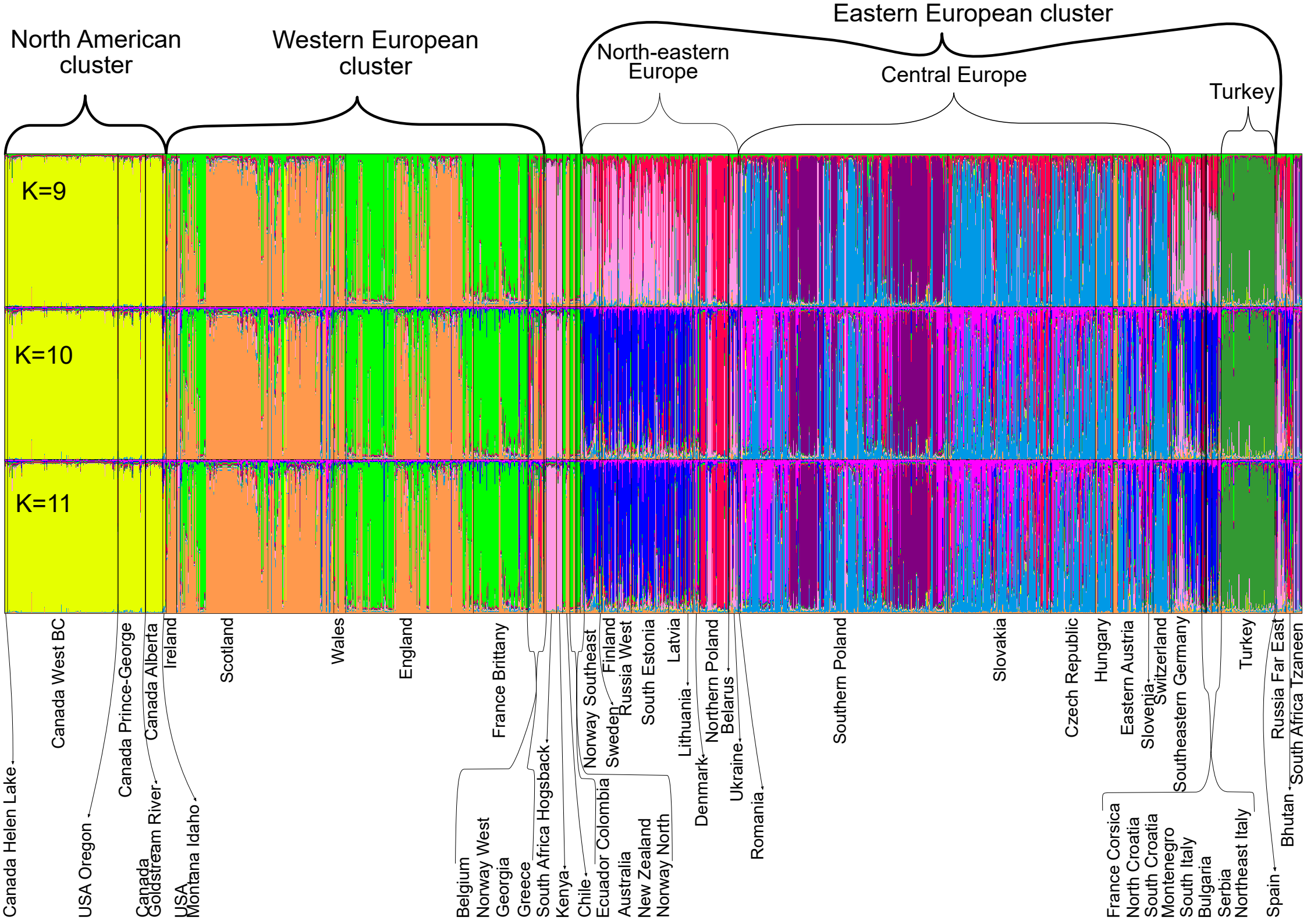

Supplement: Supplementary file 1 [file jof-07-00111-s001.zip › SupFig2bStructureK9to11.pdf]

**Value of BIC  
versus number of clusters**

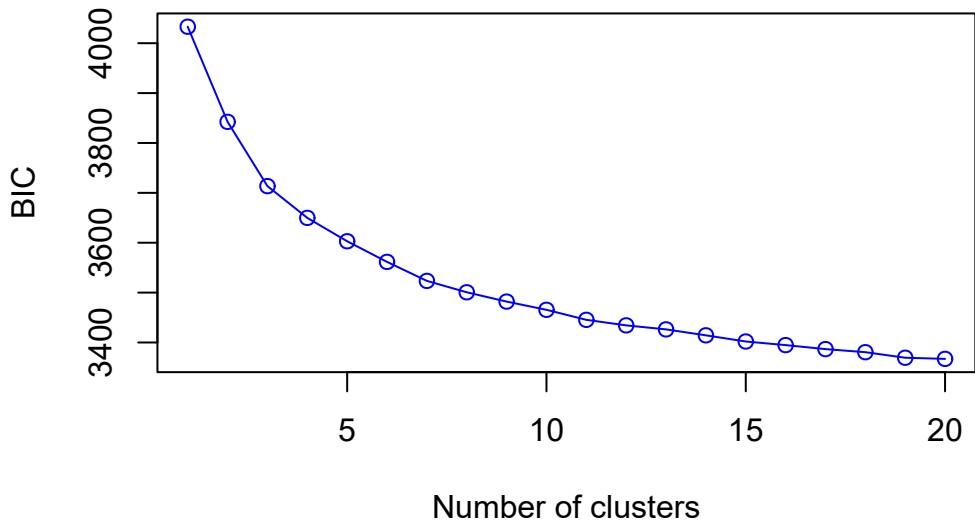

Supplement: Supplementary file 1 [file jof-07-00111-s001.zip › SupFig3 BICplot.pdf]

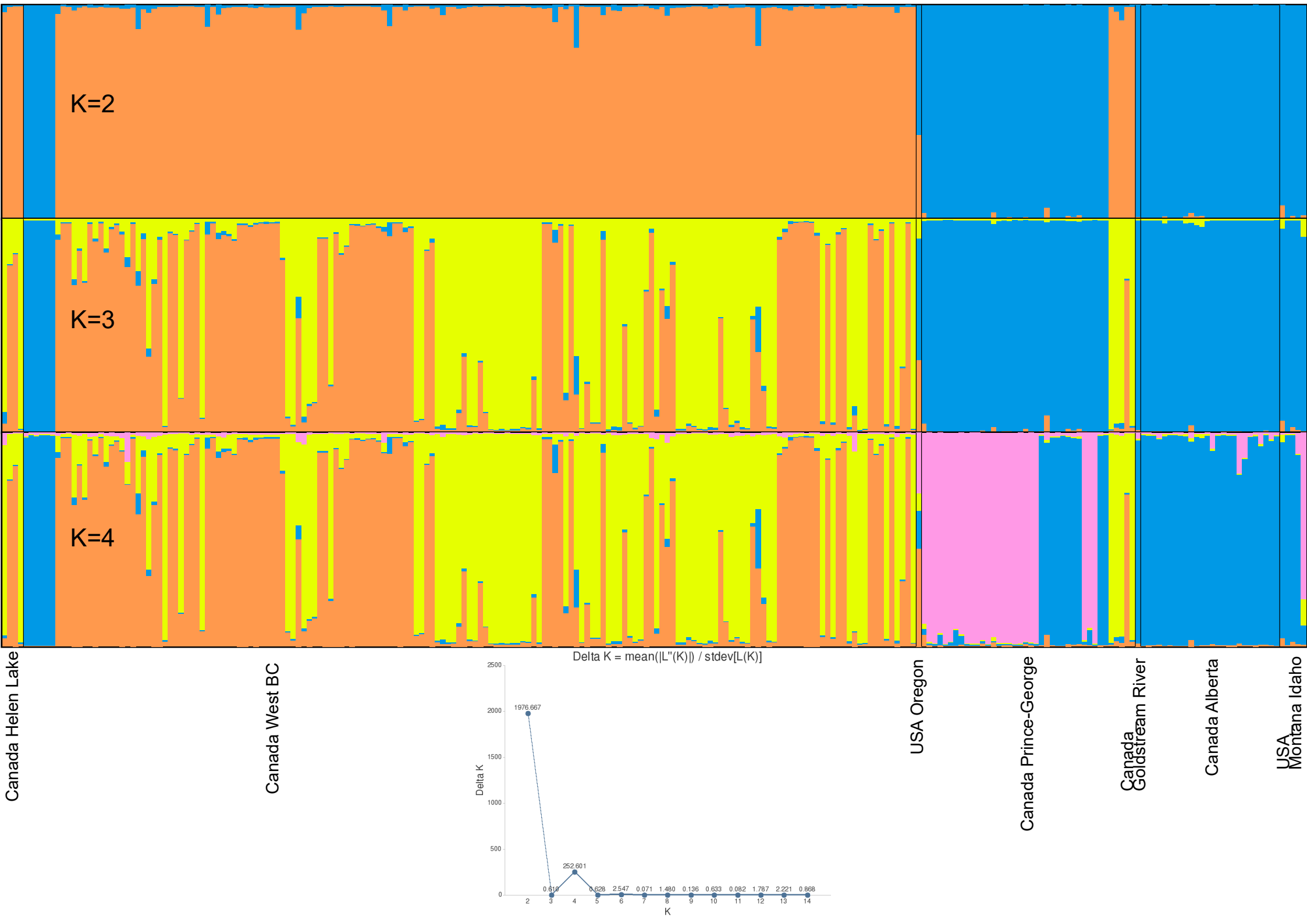

Supplement: Supplementary file 1 [file jof-07-00111-s001.zip › SupFig4 K2_3_4_NA.pdf]

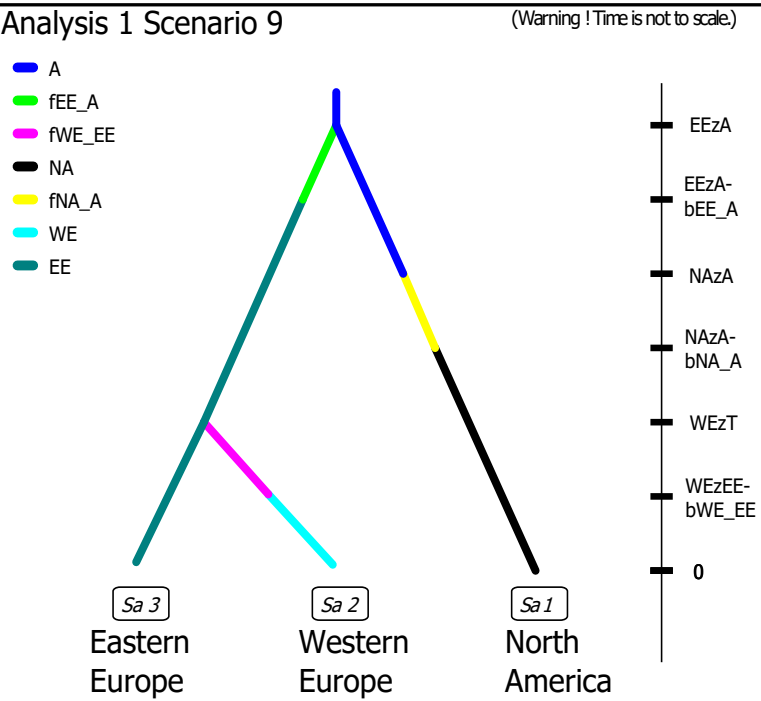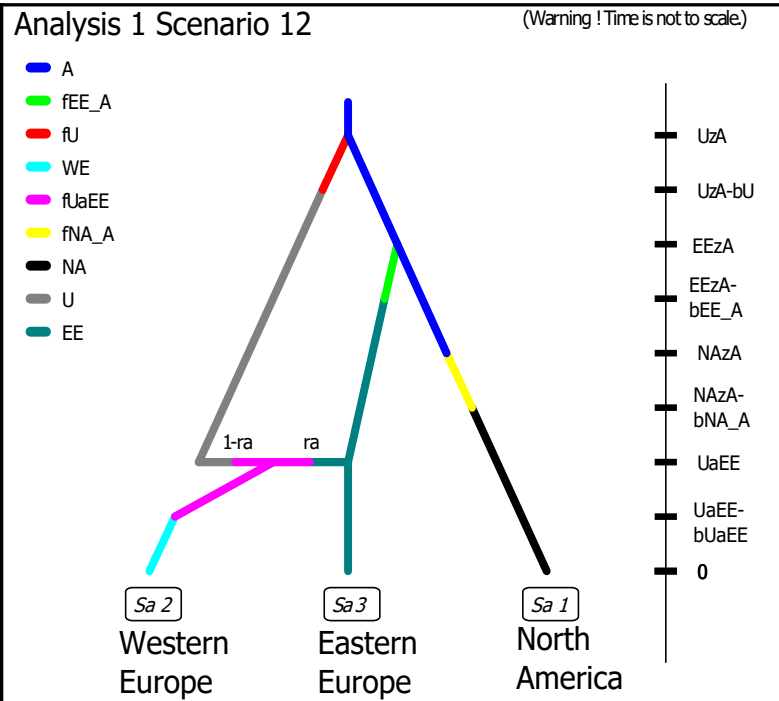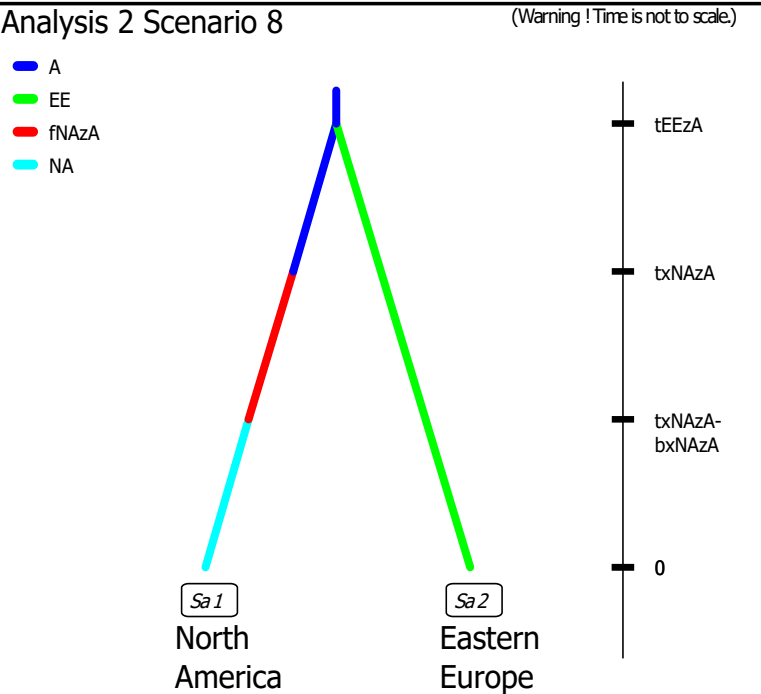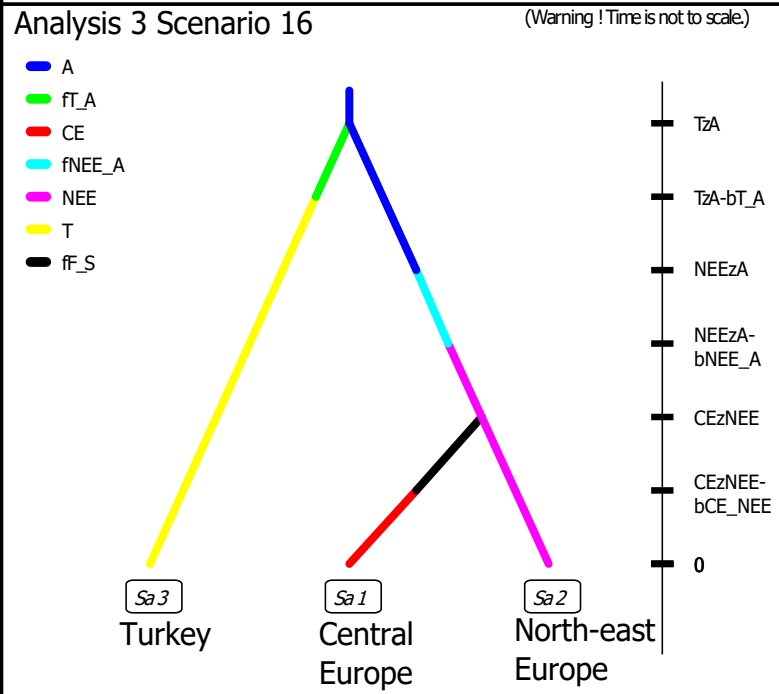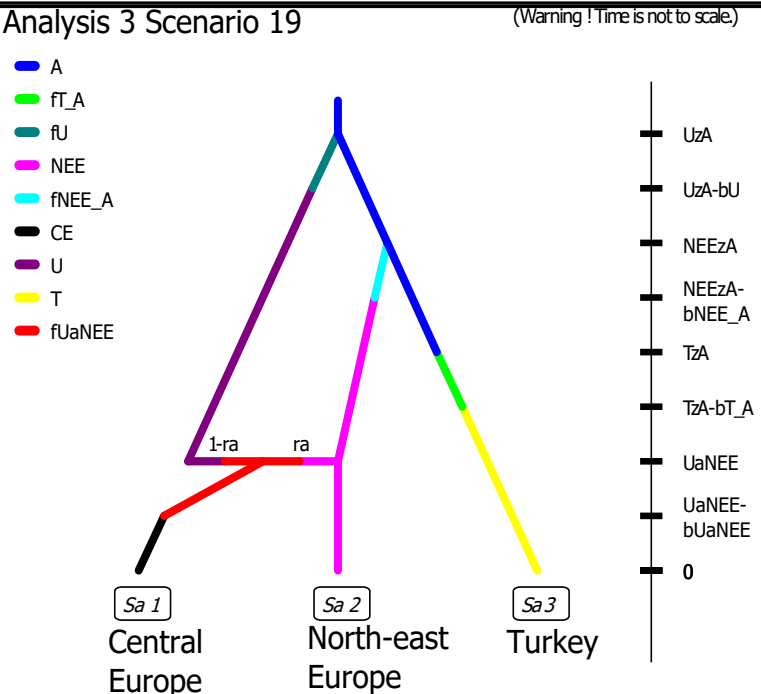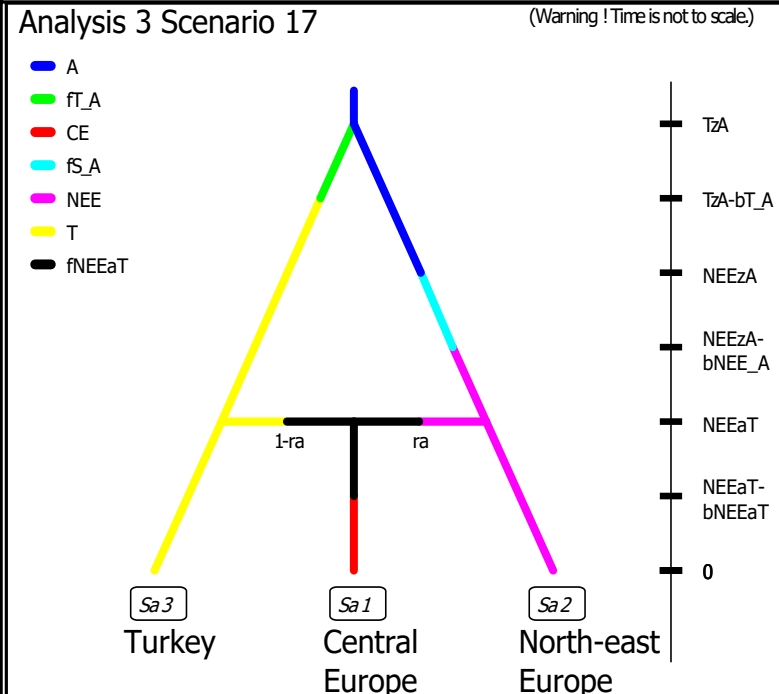

Supplement: Supplementary file 1 [file jof-07-00111-s001.zip › SupFig5 part1A1toA3.pdf]
